# Unique Configurations of Compression and Truncation of Neuronal Activity Underlie l-DOPA–Induced Selection of Motor Patterns in *Aplysia*

**DOI:** 10.1523/ENEURO.0206-17.2017

**Published:** 2017-10-24

**Authors:** Curtis L. Neveu, Renan M. Costa, Ryota Homma, Shin Nagayama, Douglas A. Baxter, John H. Byrne

**Affiliations:** Department of Neurobiology and Anatomy, W. M. Keck Center for the Neurobiology of Learning and Memory, McGovern Medical School at the University of Texas Health Science Center at Houston, Houston, TX 77030

**Keywords:** Aplysia, central pattern generator, l-DOPA, voltage-sensitive dye

## Abstract

A key issue in neuroscience is understanding the ways in which neuromodulators such as dopamine modify neuronal activity to mediate selection of distinct motor patterns. We addressed this issue by applying either low or high concentrations of l-DOPA (40 or 250 μM) and then monitoring activity of up to 130 neurons simultaneously in the feeding circuitry of *Aplysia* using a voltage-sensitive dye (RH-155). l-DOPA selected one of two distinct buccal motor patterns (BMPs): intermediate (low l-DOPA) or bite (high l-DOPA) patterns. The selection of intermediate BMPs was associated with shortening of the second phase of the BMP (retraction), whereas the selection of bite BMPs was associated with shortening of both phases of the BMP (protraction and retraction). Selection of intermediate BMPs was also associated with truncation of individual neuron spike activity (decreased burst duration but no change in spike frequency or burst latency) in neurons active during retraction. In contrast, selection of bite BMPs was associated with compression of spike activity (decreased burst latency and duration and increased spike frequency) in neurons projecting through specific nerves, as well as increased spike frequency of protraction neurons. Finally, large-scale voltage-sensitive dye recordings delineated the spatial distribution of neurons active during BMPs and the modification of that distribution by the two concentrations of l-DOPA.

## Visual Abstract

**Figure:**
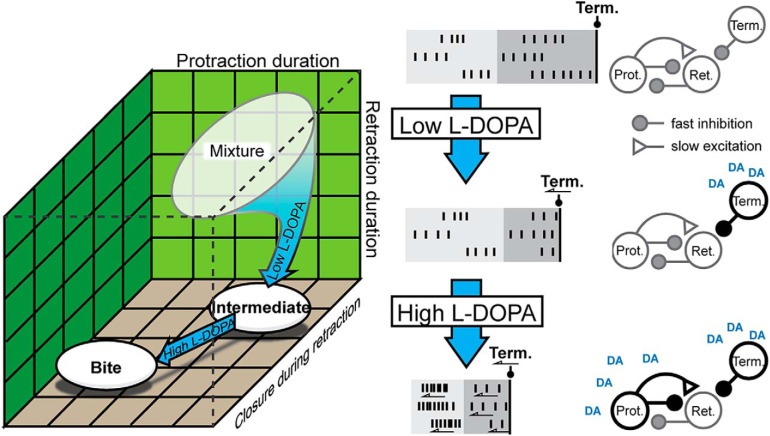

## Significance Statement

A key issue in neuroscience is understanding the ways in which neuromodulators such as dopamine (DA) modify neuronal activity to mediate selection of distinct motor patterns. We examined DA modulation of the *Aplysia* feeding motor network using l-DOPA to activate DA pathways and a voltage-sensitive dye to record activity in up to 130 neurons per preparation. l-DOPA biased selection toward distinct motor patterns and differentially modified neuronal activity in a concentration-dependent manner. DA modulation of the *Aplysia* feeding central pattern generating network may help to understand DA modulation of more complex networks in the vertebrate CNS.

## Introduction

Dopamine (DA) is considered to be a ubiquitous modulator of neuronal networks (e.g., Schultz, 2013; Wise, 2004). A great deal is known about the cellular and molecular mechanisms of DA modulation (Beaulieu and Gainetdinov, 2011) and about DA modulation of the activity of small central pattern generating (CPG) networks such as the 13-neuron lobster pyloric network (Harris-Warrick et al., 1998). However, little is known about DA modulation of individual neuronal activity of larger networks with the ability to select among many complex motor pattern outputs (Wise, 2004; Frigon, 2012; Schultz, 2013; Sharples et al., 2014). Investigating such modulation requires monitoring activity in large numbers of individual neurons with high spatiotemporal resolution.

To examine the effects of DA modulation of a relatively complex network, we simultaneously monitored the activity of up to 130 neurons in the feeding circuit of *Aplysia* using voltage-sensitive dye (VSD) imaging combined with extracellular nerve recordings. The combination of VSD and nerve recordings allowed us to record activity in individual neurons, track axonal projections, and monitor fictive motor output, enabling us to bridge the gap between individual neurons and the output of a relatively complex neuronal network. The feeding circuit, which resides primarily in the buccal ganglia, mediates several distinct behaviors, such as biting and swallowing of food and rejection of inedible objects, and generates fictive versions of these behaviors when the ganglia are isolated from the animal (Wu et al., 1988; Elliott and Susswein, 2002; Cropper et al., 2004; Baxter and Byrne, 2006; Nargeot and Simmers, 2012). Dopaminergic neurons within the buccal ganglia facilitate the genesis of buccal motor patters (BMPs) and bias the selection toward distinct BMP types (Rosen et al., 1991; Teyke et al., 1993; Kabotyanski et al., 1998; Nargeot et al., 1999b; Jing and Weiss, 2001; Díaz-Ríos et al., 2002; Due et al., 2004; Díaz-Ríos and Miller, 2005, 2006; Proekt et al., 2004; Dacks and Weiss, 2013). Although DA-induced changes of a small number of neurons have been characterized (Kabotyanski et al., 2000), there is no characterization of the circuit-wide changes induced by DA. To study the changes induced by DA in isolated buccal ganglia, we bath-applied either low or high concentrations (40 or 250 µM) of the DA metabolic precursor l-3,4-dihydroxyphenylalanine (l-DOPA), which enhances the release of endogenous DA with physiologically relevant localization and timing (Pothos et al., 1996; Kabotyanski et al., 2000; Abe et al., 2015). We found that treatment with a low concentration of l-DOPA biased motor activity toward intermediate BMPs, whereas treatment with a high concentration of l-DOPA biased motor activity toward bite BMPs. We used this concentration-dependent selection of BMPs and VSD imaging to characterize the ways in which different levels of DA modulate neuronal activity to select motor patterns.

## Methods

### Optical and electrophysiological recording

*Aplysia californica* (20–45 g) were obtained from the University of Miami National Resource for *Aplysia*. *Aplysia* are hermaphroditic. Animals were housed in plastic containers inside aerated tanks containing artificial seawater (ASW; Instant Ocean; Aquarium Systems) maintained at 15°C. Animals were fed a ∼5 × 3 cm (∼0.08 g) piece of seaweed three times per week. Animals were anesthetized by isotonic MgCl_2_ (360 mM) with a volume in milliliters equal to half the animal’s body weight in grams. The buccal mass was removed and placed in a Sylgard-lined dissection chamber containing ASW with a high (2.3×) concentration of divalent ions [330 mM NaCl, 10 mM KCl, 90 mM MgCl_2_(6H_2_O), 20 mM MgSO_4_, 30 mM CaCl_2_(2H_2_O), 10 mM HEPES, pH 7.5], which suppressed all visible movement of the buccal mass. The buccal ganglia and long segments of the peripheral nerves were isolated from the buccal mass and pinned down (caudal side facing upwards) in a Sylgard-lined imaging chamber with seven custom-made suction electrodes fastened radially. The imaging chamber was filled with normal ASW 450 mM NaCl, 10 mM KCl, 30 mM MgCl_2_(6H_2_O), 20 mM MgSO_4_, 10 mM CaCl_2_(2H_2_O), 10 mM HEPES, pH 7.5] maintained at room temperature (∼23°C) throughout the experiment with no perfusion of the saline to avoid bath agitation. An Olympus BX50WI upright microscope was equipped with a 20× 0.95-NA XLUMPLFLN water immersion objective (Olympus). The preparation was stained for 7 min in ASW containing a high concentration of RH-155 (0.25 mg/ml, AnaSpec), then the bath was exchanged with a lower concentration of RH-155 (0.025 mg/ml) and remained in this solution for the entire experiment (Hill et al., 2012). Preliminary experiments indicated that 0.25 mg/ml RH-155 yielded a greater signal-to-noise ratio than 0.1 and 0.05 mg/ml and showed no signs of toxicity. A lamp housing was fitted with a 150W halogen lamp and powered by a Kepco power supply. The light was passed through a 710/40 bandpass filter (BrightLine) and a 0.8-NA Olympus condenser. The light was then transmitted through the preparation and directed at a 128 × 128 CMOS camera (NeuroCMOS-DW128, RedShirtImaging) sampling at 2.5 kHz with a 12 Me^–^ well depth. Motor pattern generation was enhanced by a 15-s phasic stimulus (0.5 ms, 10 Hz, 100 V; WPI stimulus isolator 1850A) to buccal nerve 2 immediately before recording nerve and VSD signals for 2 min. The buccal ganglia are a symmetric pair connected by a commissure; thus, each preparation contained a pair of ganglia, one of which was selected for optical recording. For pharmacological treatment, each preparation received a 100-µL bolus of saline with either ascorbic acid [vehicle (Veh)] alone or l-DOPA (Tocris) and ascorbic acid, in close proximity to the ganglia, making a final l-DOPA bath concentration of 40 μM (low) or 250 μM (high). Treatment was administered 15 min before the posttest recording and immediately after bath exchange of the lower concentration of RH-155. The treatment remained in the bath until the end of the experiment, and each preparation received only a single treatment. The experiment was designed such that the experimenter was blind to the treatment; practically, however, this was difficult to achieve because of the dramatic changes in activity induced by l-DOPA.

### Classification of BMPs

**Figure 1.**
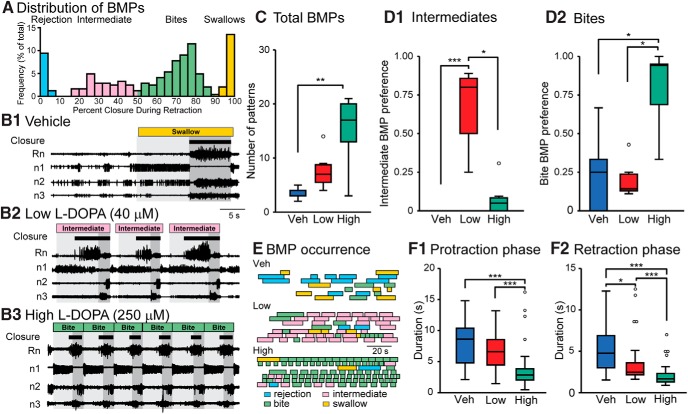
Changes in fictive behavior 15 min after treatment with Veh, 40 μM (Low), or 250 μM (High) l-DOPA. Each preparation only received a single treatment, which remained in the bath for the duration of the recording. ***A*,** Histogram of all BMPs recorded in all 21 experiments during the pretreatment observation period and following treatment. Each bin is indicated by a percentage value, calculated by dividing the duration of large-unit activity in Rn that occurred during the retraction phase by the total duration of large-unit Rn activity during the BMP. This graph indicates that there are four distinct clusters of BMPs that we designate as rejection, intermediate, bites, and swallows. ***B*,** Nerve recordings for the vehicle, low, and high treatments showing a 40-s time segment. Protraction phase is marked by light gray and retraction is marked in dark gray. The BMP classification is indicated at the top and closure activity is marked by brown boxes. Black bars under the BMP designation represent large-unit Rn activity, which has been associated with closure of the radula (Morton and Chiel 1993a,b). ***C*,** Summary data for the total number of BMPs. For all box plots, the boundaries of each box are the first and third quartiles (Q_1_ and Q_3_) and the line within the box is the median. The upper and lower extremes are the minimum (or maximum) data value within (Q_1_ or Q_3_) ± 1.5 times the interquartile range. Data outside the extremes are marked as open circles. ***D1*,** Summary data of the preference for intermediate BMPs. A value of one indicates the group exclusively expresses intermediate BMPs. ***D2*,** Summary data of the preference toward bite BMPs. Sample size for ***C***, ***D1***, and ***D2*** is seven experiments for each group. ***E*,** The occurrence of BMPs. Each box represents the duration of a single BMP. Each row is an individual experiment. ***F1*,** Duration of protraction for the different treatment groups. ***F2*,** Duration of retraction for the different treatment groups. Sample size for the groups in ***F*** is Veh = 24, Low = 54, High = 104 BMPs for seven ganglia in each group. The same dataset was examined for all subsequent figures. *p < 0.05, **p < 0.01, and ***p < 0.001.

BMPs were monitored by extracellular suction electrode recordings of ipsi- and contralateral buccal nerves 1, 2, and 3 (n1, n2, and n3), and closure activity was monitored by recording either ipsi- or contralateral radula nerve 1 (Rn; Fig. 1*A*). The start of protraction phase was considered to be the beginning of activity in n1, and the start of retraction phase was considered to be the end of activity in n1. This nerve is active during protraction (Morton and Chiel, 1993a,b) and silent during retraction and has a larger diameter than the intrinsic 2 nerve (I2n) that mediates protraction movement. Although activity in n1 may occasionally precede activity in I2n, n1 serves as a good correlate for the protraction phase. The end of retraction phase was considered to be the end of activity in n2. For Rn, large-unit activity was defined as spikes with a greater amplitude than the smallest Rn spike occurring during protraction (Nargeot et al., 1999a). In previous experiments, simultaneous intracellular and nerve recordings indicated that B8 activity corresponds to this amplitude of spikes in Rn (data not shown). Similar to previous studies, the *in vitro* preparations expressed four distinct BMP types: rejections, intermediates, bites, and swallows (Wu et al., 1988; Kabotyanski et al., 2000; Elliott and Susswein, 2002; Cropper et al., 2004; Baxter and Byrne, 2006; Nargeot and Simmers, 2012). Each of these motor patterns have been observed during *in vivo* studies of feeding behaviors (Morton and Chiel, 1993a,b). Consistent with these findings, a histogram of the distribution of BMPs with the overlap of Rn activity with the retraction phase along the *x*-axis has four reasonably distinguishable peaks (Fig. 1*A*). We categorized BMPs by setting boundaries at each trough of the histogram. BMPs with <10% of closure activity overlap with retraction were classified as rejections, 10%–50% overlap were intermediates, 50%–90% were bites, and 90% or more overlap were swallows.

### Analysis of VSD imaging data

Regions of interest (ROIs) were drawn manually by a blinded observer around each cell with Fiji (Schindelin et al., 2012), using the image frame that had the smallest mean-squared distance from the average of all frames in the recording. VSD signals were acquired by averaging the pixels in the ROI. The ROI was shifted to correct for movements of each cell that occurred during the recording. All Matlab codes can be found at www.uth.tmc.edu/byrne-lab. The raw VSD signals were bandpass filtered in Matlab (Butterworth, Fpass1 = 15 Hz, Fstop1 = 0.1 Hz, Fpass2 = 140 Hz, Fstop2 = 1 kHz, Apass = 0.1, Astop1 = 60, Astop2 = 60).

Action potentials were detected in the VSD recording data using a variation of the slope threshold method. An action potential was detected if the trace had a downward 4-ms deflection (depolarization) with an amplitude >2.5 times the SD followed 4.8 ms later by an upward deflection (measured from the downward peak) with an amplitude >3.0 times the SD. These time points were chosen because this approximated the shape of a typical action potential in *Aplysia.* This method would miss atypical action potentials generated by plateau-generating neurons such as B51 (Plummer and Kirk, 1990). A minimum separation between spikes was set to 5.2 ms to prevent counting a single spike more than once. We chose this method of spike detection because it is not computationally intensive and is resistant to changes in baseline.

### Analysis of extracellular nerve activity

**Figure 2.**
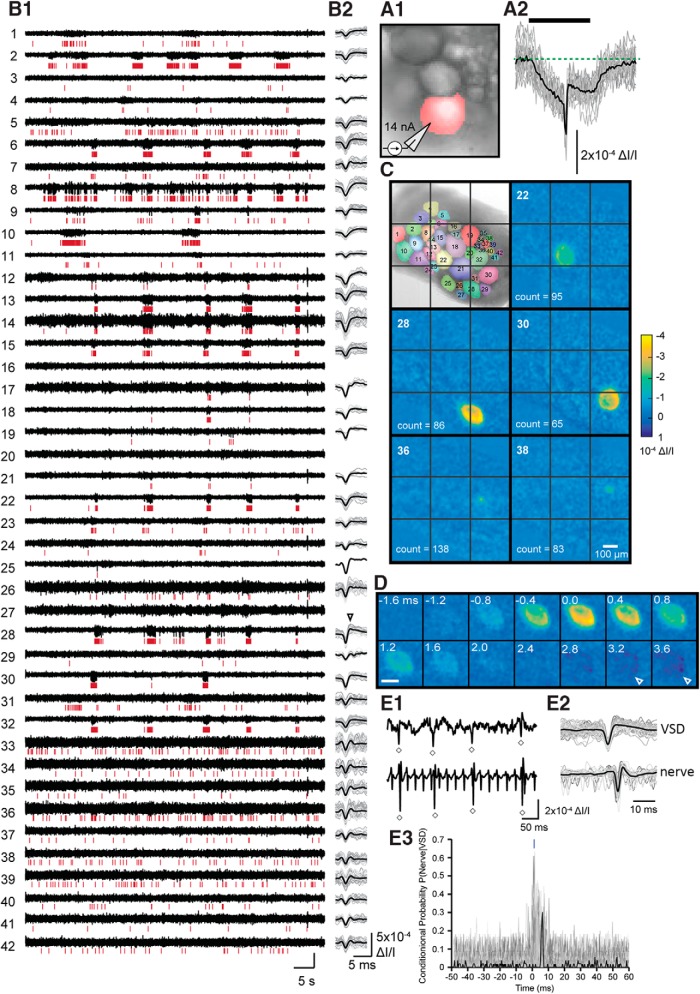
VSD imaging of neuronal activity in the buccal ganglia. ***A1*,** Image of the caudal surface of the ganglion for ***A2***. The pixels that were averaged are highlighted in red. ***A2*,** VSD responses of neuron B4 to 20 intracellular current injections, aligned to the action potential detected in the nerve (n3). Green dotted line marks the baseline before current injection. For ***A2***, ***B2***, and ***E2***, individual examples are gray and the average trace is black. Thick black line indicates the current injection. ***B1*,** VSD (black trace) of 42 neurons recorded simultaneously. Detected spikes are indicated by red vertical lines below each trace. Each trace was generated by averaging the pixels highlighted for each neuron in the top left image of ***C***. ***B2*,** Temporally aligned action potentials detected in the corresponding trace in ***A1***. Note the prominent AHP in cell 28 (arrowhead). ***C*,** Image of VSD response during the peak of the wave form of all the detected spikes that occurred in ***B*** for that neuron. The neuron designation is in the top left of each image. Image of the ganglion (caudal surface) with the neuron designations is the top left image. The number of spikes averaged for each neuron is indicated in the bottom left-hand corner. ***D*,** Individual frames of neuron 28 during and after an action potential. Arrowheads point to the presumed AHP. Data in ***A*** and ***B***, ***C***, and ***D*** are from separate animals. ***E*,** Coincidence of spikes between the nerve and neuron indicated the presence of an axonal projection. ***E1*,** Example recording segment of a neuron whose action potentials detected by VSD (top trace) coincide with a distinct spike in the nerve (bottom trace). ***E2*,** VSD traces (top) and nerve traces (bottom) of example in ***E1*** aligned by the peak of the VSD signal. Note the nerve spike follows the VSD spike with a constant delay. ***E3*,** Probability of an action potential in the nerve given a spike in the neuron at time 0. Displayed is every neuron–nerve pair for all experiments in this study. A sharp peak in conditional probability with a time delay of a few ms indicates an axonal projection. Dark line represents the example in ***E1***. Includes data from 21 preparations.

**Figure 3.**
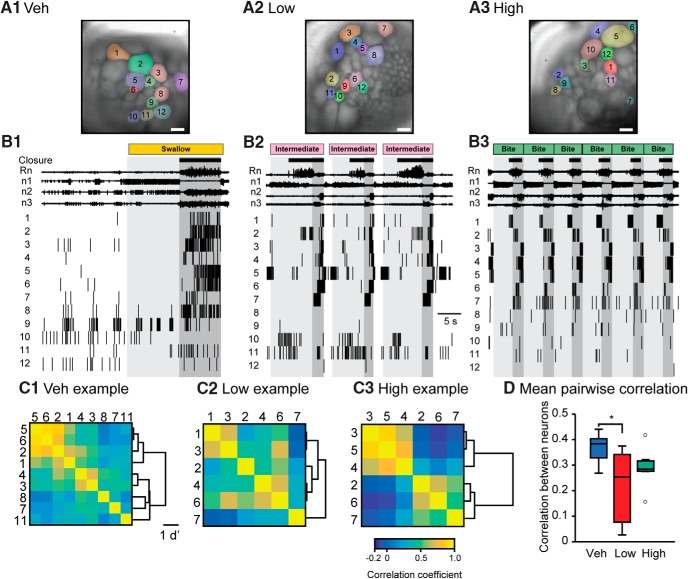
Changes in fictive behavior and neuronal activity 15 min after treatment with Veh, Low, or High l-DOPA. Each preparation received only a single treatment. Each treatment group consisted of seven preparations. A 40-s segment is shown. Recording segment same as for Fig. 1. ***A*,** Images of the ganglion (caudal surface). Neurons whose activity is shown in ***B*** are highlighted. Scale bar is 100 μm. ***B*,** Top, recordings of Rn and n1–3 nerve activity. Bottom, raster of VSD activity recorded simultaneously with nerve activity. Each row is an individual neuron with the location in the image marked in ***A***. Vertical black lines indicate an action potential. Light gray indicates protraction. Dark gray indicates retraction. Closure activity is marked by black boxes. The BMP classification is indicated at the top of the traces. ***C*,** Correlation matrices for the recordings in ***B***. Numbers correspond to the cell designations in ***A*** and ***B***. Only neurons primarily active during retraction are shown in the correlation matrix. The dendrogram of each matrix is shown on the right. ***D*,** Mean pairwise correlation between neurons. Sample sizes were seven preparations for each treatment group in panel D. Time bin was 0.5 s.

**Figure 4:**
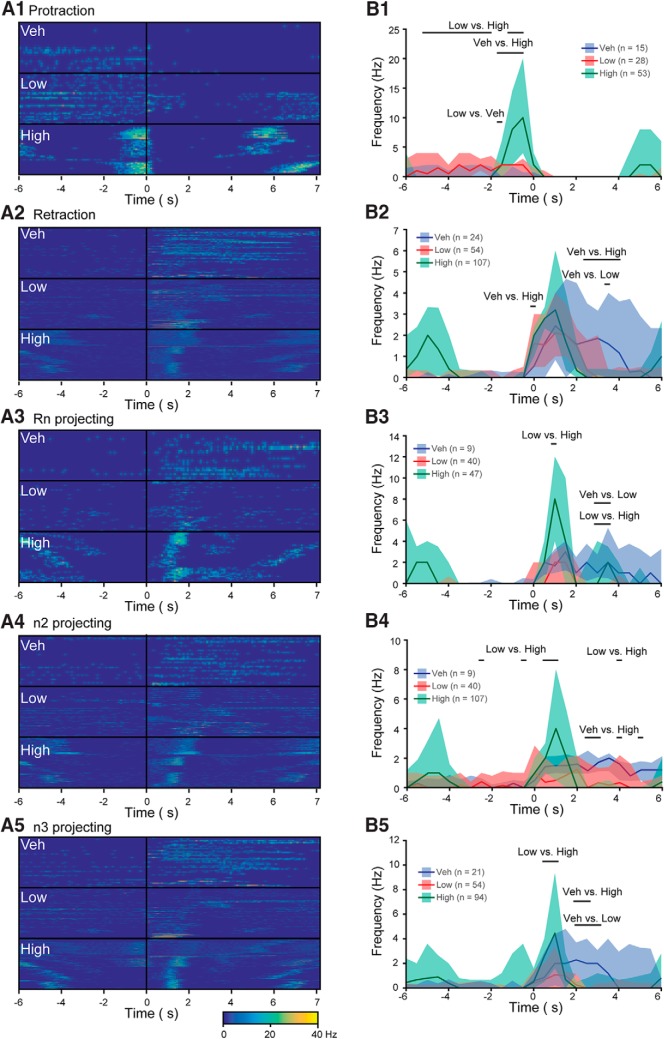
Temporal dynamics of specific groups of neurons are modified by l-DOPA. ***A*,** Activity of each neuron during each BMP aligned to the start of retraction phase. The level of activity was measured for protraction (***A1***), retraction (***A2***), and Rn (***A3***), n2 (***A4***) and n3 (***A5***) projecting neurons following treatment with Veh, Low, or High l-DOPA. Each row of the image represents the activity of a single neuron during a single BMP. The vertical black line indicates the start of retraction. The horizontal black line separates the treatment groups. Time bin was 0.1 s. ***B*,** Peri-event histograms for the data in ***A***. Horizontal bars indicate a significant difference between the specified groups at those time points. See Figures 4-1, 4-2, 4-3, 4-4 and 4-5 for statistics on ***A1***, ***A2***, ***A3***, ***A4*** and ***A5***, respectively. Peaks in activity outside ± 4 s for High are due to activity in adjacent BMPs. The fill represents the interquartile range and the line represents the median level of activity of each time point. Time bin was 0.5 s. Sample size is number of BMPs. Each treatment group consisted of seven preparations.

**Figure 5:**
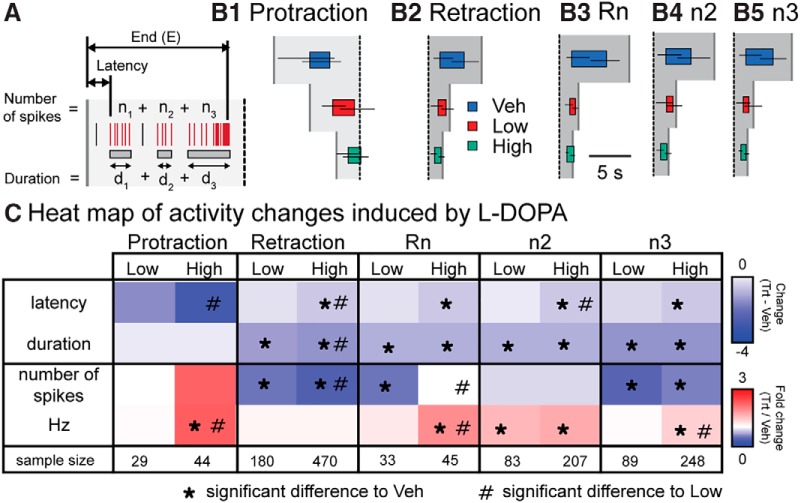
l-DOPA differentially modified the burst properties of neurons. ***A*,** Diagram depicting the measurements. The latency is the start of the first burst overlapping with the phase. The duration is the sum of the duration of all bursts for that neuron during the phase. The burst end time is the end of the last burst overlapping with the phase. The number of spikes is the sum of spikes within all the burst overlapping that phase. Frequency is the number of spikes divided by the burst duration. ***B*,** Graphical representation of the burst timing during a BMP. The colored box represents the median start and end time for bursts in that treatment. The horizontal lines represent the interquartile range for the start and end time of the bursts. The vertical gray lines in ***B1*** indicate the median start of protraction. The vertical gray lines in ***B2–B5*** indicate the median end of retraction. The vertical dotted line indicates the start of retraction. ***C*,** Matrix of the l-DOPA induced changes in burst times and spiking activity. For display purposes, Veh median start and duration were subtracted from the median start and duration of either Low or High. For spiking activity, the number or frequency of spikes was divided by the median number or frequency of spikes in Veh. *, Significance relative to Veh; #, significance relative to Low. See Fig. 5-1 for statistics. The sample size is the number of bursts examined. Each treatment group consisted of seven preparations.

**Figure 6.**
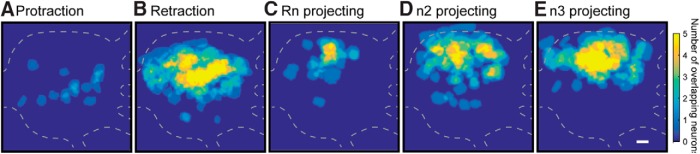
Spatial distribution of protraction, retraction, Rn, n2, and n3 projecting neurons. ***A*,** Protraction neurons are clustered near the center. ***B*,** Retraction neurons are near the upper middle. ***C*,** Rn projecting neurons are in the center upper region. ***D*, *E*,** n2 and n3 neurons are in the topmost region of the ganglia. Caudal surface with the buccal commissure on the left. Scale bar is 100 μm. Data were pooled across all 21 preparations.

**Figure 7.**
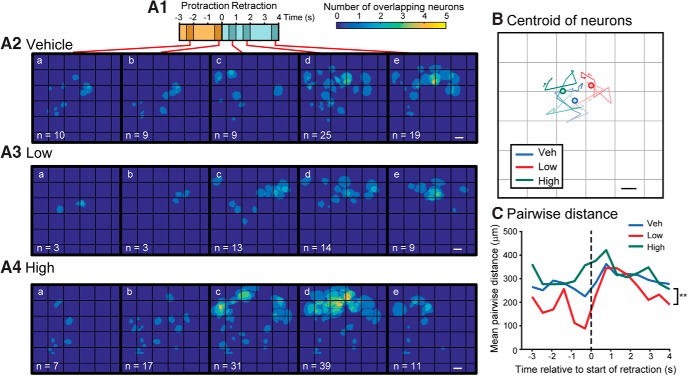
l-DOPA tended to recruit neurons with different spatial distributions. Data were pooled across all seven preparations for each treatment group. ***A1***, Timeline of a BMP. ***A2*–*A4*,** Images of the locations of all neurons activated at the time bins indicated in ***A1*** from all experiments within a given treatment. Five of the 14 time bins are shown. The pixels for each overlaying ROI were summed. The number at the bottom left is the number of neurons activated. Caudal surface with the buccal commissure on the left. Scale bar is 100 μm. ***B*,** The centroid of all neurons as it progresses through each time bin in ***A1***. An opacity gradient was added to the line to represent time (light→dark represents beginning→end). The filled circle marks the mean of all the centroids. ***C*,** Mean pairwise distance between all pairs of neurons for each time bin. **p < 0.01.

The voltage from the extracellular nerve electrodes was amplified by a differential AC amplifier (A-M Systems 1700) and digitized by the A-D converter of the CMOS camera system. The raw extracellular voltage signals were low-pass filtered in Matlab (equiripple, Fpass = 200 Hz, Fstop = 1 kHz, Apass = 1, Astop = 60, stopband shape = flat). The waveforms of action potentials in the nerve had a variety of shapes (Fig. 2*E1*). Therefore, we used a similar spike detection method as for VSD imaging, but with several differences. Nerves had a high baseline activity (e.g., n2 in Fig. 1*B1*); thus the spike detection was run in 5 iterations to remove spikes to gain a more accurate estimation of SD of the noise. The threshold for the initial downstroke was 3.0 and the upstroke was 3.5 times the SD, which was calculated after the spikes were zeroed out from the previous iteration using a 4-ms before/6.8-ms after time window. The width of the spike had to fit one of three empirically determined durations, {1.2/1.2}, {2.0/2.0}, {3.2/2.8}, where the notation is {[duration of downstroke]/[duration of upstroke]} in milliseconds. In addition, the polarity of the spikes sometimes alternated between preparations; therefore, the data were also scanned for the inverse wave form (except the {2.0/2.0} criterion, whose inverse was not included because it had a large number of false positives). The spike times of the final iteration were used for the identification of axonal projections (see below). The multiple criteria were needed to increase the performance of the spike detection algorithm and allow the detection of spikes with different waveforms. The performance of the spike detection of the nerve recordings was confirmed by visual inspection. The parameters for VSD and nerve spike detection were optimized and fixed before starting the subsequent analyses presented in Figs. 3–7.

### Identification of axonal projections from neurons to nerves

Spike coincidence between each nerve and each neuron was measured by first calculating the probability of an action potential in the nerve given an action potential in the neuron (Fig. 2*E3*) and then subtracting the mean probability of an action potential in the nerve 0–50 ms before the action potential in the neuron, P(nerve | neuron) – P(nerve). P(nerve | neuron) was calculated by summing the probability of an action potential in the nerve within a window around (2 ms before/2 ms after) the highest peak in probability with a positive delay (see peak in Fig. 2*E3*). P(nerve) estimates the level of activity of the nerve around the same time as the spikes in the neuron. To identify an axonal projection, spike coincidence was required to be at least 0.25.

### Spike correlation

The recording for each retraction neuron was binned into 0.5-s segments, and the spike frequency was calculated for each time bin. The *corr* function in Matlab was used to calculate Pearson’s linear correlation coefficient between each pair of retraction neurons in each preparation. The correlation matrix was then clustered using the linkage followed by the cluster and dendrogram functions in Matlab. The maximum number of clusters was set to 4.

### Burst analysis

A burst was considered to be a series of at least three spikes (Cocatre-Zilgien and Delcomyn, 1992) with a maximum interspike interval of 400 ms (Chiappalone et al., 2005). To remove neurons with a high baseline firing rate that by chance may meet this threshold, bursting neurons were required to have a substantial difference in spike frequency within bursts compared to outside of bursts (as indicated by Fisher’s exact test; Cocatre-Zilgien and Delcomyn, 1992). Fisher’s test was made more conservative by dividing the spike frequency within bursts by four.

Bursting neurons were grouped into those activated primarily during protraction or retraction phase. A cell was considered to be primarily active during either protraction or retraction when at least 75% of its burst activity overlapped with the respective phase. Neurons that shifted phase between recordings were rarely observed. Therefore, the posttest recordings and a 2-min pretest observation period were combined to improve the classification. To confirm the accuracy of our classification, this procedure was applied to 98 published recordings of identified neurons provided in the literature (Church and Lloyd, 1994; Borovikov et al., 2000; Jing and Weiss, 2001; Shetreat-Klein and Cropper, 2004; Sasaki et al., 2009; Bédécarrats et al., 2013; Sieling et al., 2014). The activity of each neuron was obtained by using the snapshot tool in Adobe Acrobat X to capture an image of the data and Matlab to convert the pixelated images of the published recordings to spike trains. In 98% of the examples, our classification matched what was specified in the literature, indicating that our method agrees with the general consensus.

Bursts were considered to be associated with a given BMP if for protraction neurons the burst overlapped with the protraction phase. For retraction, Rn, n2, or n3 neurons, the burst was associated with a BMP if the burst overlapped with the retraction phase, because this was the phase in which these neurons were primarily active (see Fig. 5). Burst latency was calculated as the delay between the start of the first burst of activity in the neuron and the start of the phase for each BMP. The duration was calculated for each BMP by summing the duration for all bursts in the neuron that overlapped with the phase.

### Topographical analysis

Each ganglion was aligned to a universal grid. The orientation of each ganglion was approximated by calculating the mean slope and offset of the ventral neuron cluster and the ganglion as a whole relative to the universal grid. A linear regression was performed on the coordinates of all the pixels of all the neurons having a large area (the largest 50% of neurons in the field of view). These large neurons primarily reside in the ventral neuron cluster, which is composed of large motor neurons that run parallel to the longitudinal axis of the ganglion. The pixels that overlay the entire ganglion were observed to have a light intensity between the 5th and 90th percentiles of all the pixels within the field of view. Therefore, to improve the estimation of the ganglion orientation, a second linear regression was performed on the pixel coordinates within this range of intensities. The mean slope and offset of these two regression lines relative to the universal grid approximated the orientation of the ganglion. The image was then rotated and shifted in the *x–y* direction according to this orientation.

### Statistical analysis

All statistical analyses were performed in Matlab using the statistical toolbox (The Mathworks). Normality was not assumed for any of the analyses. For the peri-event histograms (Fig. 4) a Kruskal–Wallis test was followed by a multiple comparisons of mean ranks test with Bonferroni correction, which multiplied the *p* value by the number of time points and the number of treatment groups. For timing of burst activity analysis (Fig. 5), Kruskal–Wallis test was followed by a pairwise rank sum test with a Bonferroni correction where the *p* value was multiplied by the number of neuron subgroups and the number of treatment groups. Heteroscedasticity was not tested before the analyses. For all comparisons, a *p* value <0.05 was considered statistically significant. Superscript letters listed with *p* values correspond to the statistical tests shown in Table 1.

**Table 1.** Statistical table.

	Data structure	Type of test	Statistical value (χ^2^)	*p* value
a	Normality not assumed	Kruskal–Wallis	11.513	0.003
b	Normality not assumed	Kruskal–Wallis	11.359	0.034
c	Normality not assumed	Kruskal–Wallis	16.145	3.1 × 10^−4^
d	Normality not assumed	Kruskal–Wallis	65.499	5.9 × 10^−15^
e	Normality not assumed	Kruskal–Wallis	65.232	6.9 × 10^−15^
f	Normality not assumed	Kruskal–Wallis	1.789	0.409
g	Normality not assumed	Kruskal–Wallis	6.264	0.044
h	Normality not assumed	Kruskal–Wallis	859.92	1.3 × 10^−133^
i	Normality not assumed	Kruskal–Wallis	1575.30	4.4 × 10^−280^
j	Normality not assumed	Kruskal–Wallis	660.15	6.3 × 10^−95^
k	Normality not assumed	Kruskal–Wallis	913.19	8.1 × 10^−145^
l	Normality not assumed	Kruskal–Wallis	707.03	4.9 × 10^−104^
m	Normality not assumed	Kruskal–Wallis	0.998	0.607
n	Normality not assumed	Kruskal–Wallis	0.800	0.670
o	Normality not assumed	Kruskal–Wallis	0.236	0.889
p	Normality not assumed	Kruskal–Wallis	1.051	0.591
q	Normality not assumed	Kruskal–Wallis	1.628	0.443
r	Normality not assumed	Kruskal–Wallis	144.63	8.5 × 10^−24^
s	Normality not assumed	Kruskal–Wallis	217.68	1.3 × 10^−38^
t	Normality not assumed	Kruskal–Wallis	133.82	1.2 × 10^−21^
u	Normality not assumed	Kruskal–Wallis	165.54	5.4 × 10^−28^
v	Normality not assumed	Kruskal–Wallis	12.192	0.0023

## Results

### l-DOPA enhances specific fictive behaviors in a concentration-dependent manner

We used two concentrations of l-DOPA, denoted High and Low. The High concentration (250 μM) was identical to that used by Kabotyanski et al. (2000), which was previously shown to modulate the feeding network. Because previous studies reported concentration-dependent effects of l-DOPA (Kemnitz, 1997), we also tested whether treatment with a lower concentration of l-DOPA (Low, 40 μM) differentially modulated the feeding network. Buccal ganglia were isolated, and nerve activity was recorded with suction electrodes to monitor BMPs (see Methods). BMPs consist of two phases. The first phase is protraction, defined here as activity in n1, and the second phase is retraction, defined here as activity in n2 and absence of activity in n1 (see Methods). These phases were previously found to correspond to outward (protraction) and inward (retraction) movement of the radula, a tongue-like structure (Morton and Chiel, 1993a; Neustadter et al., 2002, 2007). Activity of the radula nerve 1 (Rn) is a correlate of closure movement *in vivo*. Greater overlap of Rn activity with retraction corresponds to a larger inward movement of food (Morton and Chiel, 1993a). Therefore, we classified BMPs into four categories based on the overlap of activity in Rn with the retraction phase (Methods and Fig. 1*A*). Two of the BMP categories resembled *in vivo* nerve activity during the ingestion of food (bite and swallow), one category resembled the activity during the rejection of food (rejection), and one category resembled nerve activity during a behavior that resulted in little to no movement of food (intermediate; Morton and Chiel, 1993a).

Each preparation received a single treatment of ascorbic acid (Veh), Low, or High l-DOPA (Fig. 1*B*). Consistent with Kabotyanski et al. (2000), High l-DOPA increased the total number of BMPs (Fig. 1*B3*,*C*; χ^2^ = 11.513, *p* = 0.003; *post hoc,* Veh vs. High, *Q* = 3.358, *p* = 0.0023; Low versus High, *Q* = 1.256, *p* = 0.420).^a^ Low l-DOPA treatment tended to increase BMPs, but this increase was not significant (Veh vs. Low, *Q* = 2.101, *p* = 0.090). Consistent with Kabotyanski et al. (2000), High l-DOPA treatment increased the preferential expression of bite BMPs (Fig. 1*B3*,*D2*; χ^2^ = 11.359, *p* = 0.034; *post hoc,* Veh vs. Low, *Q* = 0.086, *p* = 0.996; Veh vs. High, *Q* = 2.875, *p* = 0.011; Low vs. High, *Q* = 2.961, *p* = 0.0086)^b^. Low l-DOPA treatment did not increase the preference toward bites but instead increased the preference toward intermediates (Fig. 1*B2*,*D1*; χ^2^ = 16.145, *p* = 3.1 × 10^−4^; *post hoc,* Veh vs. Low, *Q* = 3.947, *p* = 2.3 × 10^−4^; Veh vs. High, *Q* = 1.323, *p* = 0.382; Low vs. High, *Q* = 2.624, *p* = 0.024)^c^. Veh seemed to express a mixture of BMPs (see Fig. 1*B1*,*E*). These data indicate that l-DOPA increased total BMPs and that different concentrations of l-DOPA can be used to bias selection toward specific BMPs.

To examine whether l-DOPA treatment modified each phase of the BMP, the duration of protraction and retraction was measured for each BMP after treatment. Protraction duration was reduced in High but not Low l-DOPA (Fig. 1*F1*; χ^2^ = 65.499, *p* = 5.9 × 10^−15,^ *post hoc*, Veh vs. Low, *Q* = 1.034, *p* = 0.555, Veh vs. High, *Q* = 6.07, *p* = 4.8 × 10^−9^, Low vs. High, *Q* = 6.692, *p* = 1.0 × 10^−9^).^d^ Retraction duration was reduced for Low (χ^2^ = 65.232, *p* = 6.9 × 10^−15^, *post hoc*, Veh vs. Low, *Q* = 2.635, *p* = 0.023)^e^ and was reduced to a greater extent by High l-DOPA treatment (Fig. 1*F2*; Veh vs. High, *Q* = 5.618, *p* = 1.0 × 10^−9^; Low vs. High, Q = 5.618, *p* = 5.9 × 10^−8^). Many neurons in the feeding circuit can be designated as protraction or retraction neurons based on the phase in which they are primarily active. The above results suggest that protraction neurons may be less sensitive to l-DOPA treatment than retraction neurons. Reduction in retraction neuron activity duration due to Low l-DOPA treatment may be important for the expression of intermediate BMPs, whereas combined reductions in protraction and retraction neuron activity duration due to High l-DOPA treatment may be important for expression of bite BMPs. This hypothesis was tested with VSD imaging of neuronal activity.

### VSD imaging captures spike activity in a large number of neurons of the buccal ganglia

To gain insight into the ways in which changes in neuronal activity mediate the changes in fictive motor programs induced by l-DOPA, the spiking pattern of neurons in the buccal ganglia was examined using the absorbance voltage-sensitive dye RH-155. This dye has been used in the pedal ganglia of *Aplysia* (Bruno et al., 2015). To confirm its efficacy in the buccal ganglia, changes in light absorbance were recorded in neuron B4 during stimulation of B4 by intracellular depolarizing current pulses (14 nA, 50 ms; Fig. 2*A*). VSD traces exhibited a prominent downward spike (increase in absorbance) resembling an action potential that was superimposed on a more sustained downward deflection resembling a depolarization induced by the current injection. The action potential was followed by a signal corresponding to the spike afterhyperpolarization.

Next, spiking activity was recorded in 20–130 neurons simultaneously over a 2-min recording period (Fig. 2*B*). Some of this activity occurred in bursts (e.g., cell 28), whereas activity in other neurons was more sparse (e.g., cell 19). We then converted the activity to spike trains using a spike detection algorithm (Methods) and verified that the VSD signals corresponding to the spikes were localized to the neuron of interest (Fig. 2*C*). Two frames were averaged during the baseline period just before the spike and subtracted from the average of 3 frames at the peak of the spike. This subtracted image was calculated for each detected spike and averaged for all spikes that occurred in that neuron during a 2-min recording period (Fig. 2*C*). The averaged subtracted image revealed a VSD signal that closely matched the shape and position of the cell for which the spikes were detected. Importantly, even recordings associated with higher levels of baseline noise (e.g., cell 36) had a signal localized to that particular neuron. Moreover, VSD recordings exhibited signals associated with presumed spike afterhyperpolarizations (AHPs) as indicated by an upward deflection after the spike (e.g., arrow in Fig. 2*B2*) and decrease in absorbance in the pixels overlaying the neuron (arrows in Fig. 2*D*, Video 1).

Video 1. VSD changes for nine neurons during an action potential. The raw images of nine cells in Fig. 2*A* are displayed with an outline of the ROI. The ROI designations are in the top left of each image. The video plays at 1/500 speed to highlight the spatiotemporal voltage response of each cell. The VSD response appears to be centralized to the ROI (also seen in image preview). An apparent AHP can be seen following the action potential (3.2–3.6 ms; not seen in image preview). The scale bar is 100 μm. Images were filtered with a 1-pixel Gaussian filter. 10.1523/ENEURO.0206-17.2017.video.1

Combining VSD with extracellular nerve recordings enables the monitoring of BMPs while also enabling the detection of axonal projections of the recorded neurons. Previous work in the buccal ganglia (Morton et al., 1991) used averaging of extracellular nerve recordings triggered by spikes detected in VSD recordings to detect axonal projections by the emergence of a wave form in the averaged trace. That method requires averaging a large number of action potentials to average out randomly occurring large-amplitude spikes. Instead, we used spike coincidence to detect action potentials in the nerve that follow an action potential in the neuron with a relatively constant delay (Fig. 2*E1*,*E2*). To obtain a quantitative method of distinguishing neurons with axonal projections, we graphed the probability of an action potential occurring in the nerve at different time delays relative to an action potential in the neuron, P (nerve | neuron). We noticed a sharp peak in probability following the action potential in the nerve. This peak was used to estimate the spike coincidence and detect an axonal projection algorithmically (Methods, Fig. 2*E3*).

These data provide evidence that imaging with high spatial and temporal resolution can record activity of a large number of neurons simultaneously in the buccal ganglia and can be used to detect axonal projections. We next examined the ways in which l-DOPA reconfigures the activity of neurons mediating the BMPs.

### l-DOPA modifies neuronal activity without increasing neuronal synchrony

As a first step, we examined the extent to which neuronal activity recorded by VSD corresponded to the phases of the BMP. For data analysis, we focused only on neurons with bursting activity (for definition, see Methods) and did not include neurons with tonic or sparse activity, because bursting neurons mediate the majority of the features observed during a BMP (Wu et al., 1988; Elliott and Susswein, 2002; Cropper et al., 2004; Baxter and Byrne, 2006; Nargeot and Simmers, 2012). There were 28.6 ± 6.0 (29.6% of total within field of view) neurons per experiment categorized as bursting in Veh, 25.5 ± 4.6 (25.5%) in Low l-DOPA, and 31.3 ± 3.2 (31.3%) in High l-DOPA, with no significant differences among the groups (χ^2^ = 1.789, *p* = 0.409).^f^ Spike activity was recorded after treatment with Veh, Low, or High. The activity of neurons occurred during specific phases of the BMP. For example, in Fig. 3*B1*, neurons 1–8 and 11 seemed to fire primarily during the retraction phase, whereas neuron 1 in Fig. 3*B3* was primarily active during the protraction phase. The VSD recordings revealed that the l-DOPA–induced changes in BMPs as monitored via nerve recordings (Fig. 1) were correlated with enhanced rhythmic activity in a large number of neurons in the buccal ganglion. Interestingly, each neuron tended to be recruited at specific times within a particular phase even when the phase was shorter in duration (e.g., in High preparations, Fig. 3*B3* and Video 2), suggesting that the synchrony of neuronal activity was not increased by l-DOPA.

Video 2. Neuronal activity after treatment with Veh, Low, or High l-DOPA. Each video segment corresponds to the activity in Veh, Low (shown in image preview), and High treatment groups. An image of the ganglia is on the left and a raster plot of the activity is on the right. The nerve activity is on the top right. The ROI overlying the neuron is highlighted when the neuron is active. Activity of each neuron is also indicated by a tone with specific pitch (the lowest tone is assigned to Neuron 1 and the highest to Neuron 12). 10.1523/ENEURO.0206-17.2017.video.2

To analyze neuronal synchrony, the correlation coefficient was examined for each pair of retraction neurons in a given preparation (Fig. 3*C*,*D*; for details, see Methods). We focused on retraction neurons for the correlation analysis because retraction neurons seemed most reliably activated during BMPs. The spike activity for each neuron was binned into 0.5-s segments, and a linear Pearson’s pairwise correlation was calculated between each pair of retraction neurons of each preparation. Pairwise correlation matrices of the examples in Fig. 3*B* revealed high correlation coefficients between several neurons for Veh (e.g., cells 2 ↔ 1, 5 ↔ 2, 5 ↔ 6), Low (e.g., cells 3 ↔ 1, 6 ↔ 3, 6 ↔ 4), and High (e.g., cells 4 ↔ 3, 5 ↔ 3, 6 ↔ 2). If the l-DOPA–induced decrease in retraction phase duration resulted in an increase in synchrony between neurons, the correlation matrix would become more homogeneous, and the mean pairwise correlation would increase. Cluster analysis of the correlation matrices of Veh, Low, and High identified several groups of neurons. For the preparation of Figs. 3*C*,*D*, these groups are indicated by the dendrograms to the right of each matrix. In the example for Veh, neurons could be separated into groups {5, 6, and 2}, {4 and 3}, and {8, 7, and 11}; the Low example could be separated into groups {1 and 3}, {2, 4, and 6}, and 7; and the High example could be separated into groups {3, 4, and 5} and {2, 6, and 7}. We next averaged the pairwise correlation coefficient for every pair of retraction neurons for each experiment and compared the mean correlation coefficient between treatments. Low l-DOPA treatment did not increase but instead decreased the mean pairwise correlation, whereas High l-DOPA led to no significant change (χ^2^ = 6.264, *p* = 0.044, *post hoc*, Veh vs. Low, *Q* = 2.498, *p* = 0.033, Veh vs. High, *Q* = 1.378, *p* = 0.352, Low vs. High, Q = 1.120, *p* = 0.502). These results indicate that l-DOPA treatment did not increase but rather decreased the synchrony of neuron activity, suggesting that the unique timing of neuronal activity remains an important feature even when the durations of the respective phases are substantially shorter. The unique timing of activity of each neuron within a given phase despite reduced phase duration highlights the intricacies of the phasic activity of the neurons within the circuit, warranting a more detailed investigation of l-DOPA–induced changes in the timing of activity of individual neurons during BMPs.

### l-DOPA reconfigures activity of specific subgroups of neurons

We next examined the ways in which the features of the BMP were associated with changes in the timing of neuronal activity during BMPs. We first separated the neurons according to their preferred phase (protraction or retraction; see Methods) and then used peri-event histograms aligned to the start of the retraction phase to compare neuronal activity among treatments. A histogram of the average activity of all neurons in each time point was calculated for each BMP in each treatment group. The activity of every bursting neuron in every BMP in each treatment is depicted in Fig. 4*A*; the summary data for all the BMPs in each treatment is depicted in Fig. 4*B*; and the results of the statistical comparisons are tabulated in Figs. 4-1 through 4-5.

10.1523/ENEURO.0206-17.2017.f4-1 Figure 4-1 Statistical comparisons among protraction neurons for Fig. 4. The treatment groups were compared for each time bin (column 1), and each comparison is represented in columns 2–4. The first row for each time bin is the *t* value. The second row for each time bin is the Bonferroni-adjusted *p* value. Bonferroni-adjusted *p* values can be larger than one; such *p* values were capped at one in this and subsequent tables. *, *p* < 0.05, **, *p* < 0.01, and ***, *p*<0.001. Kruskal–Wallis test indicated a significant difference between the groups (χ^2^ = 859.92, *p* = 1.3 × 10^−133^)^h^. Download Figure 4-1, DOC file.

We first examined protraction neurons, whose activity is correlated with the outward protraction of the radula (Susswein and Byrne, 1988; Teyke et al., 1993; Hurwitz et al., 1994, 1996, 1997; Kabotyanski et al., 1998). Because High l-DOPA yielded the greatest increase in frequency of BMPs, we predicted an increase in activity of protraction neurons in preparations treated with High l-DOPA. The peri-event histogram for protraction neurons (Fig. 4*B1*) indicated a prominent peak of activity before the start of the retraction phase, which appeared to have a substantially shorter duration in High l-DOPA (as indicated by a decrease in activity at earlier time points) and a substantially greater level of activity near the end of the protraction phase (Figs. 4*A1*,*B1* and 4-1). These data indicated that only High l-DOPA treatment increased the spike frequency and decreased the duration of protraction neuron activity, suggesting that changes in protraction neuron activity may be important for bite, but not intermediate, BMPs. These activity changes may help to explain the increase in total patterns in High l-DOPA.

The second group examined was neurons active primarily during retraction. These neurons are important for retracting the radula inward and either releasing or maintaining the grip on food (e.g., Plummer and Kirk, 1990; Church and Lloyd, 1994; Hurwitz and Susswein 1996; Evans and Cropper 1998; Cropper et al., 2004; Sasaki et al., 2013). The peak frequency of retraction neuron activity was not significantly different between the treatments, but the activity was shorter in duration in the Low and High groups compared with Veh, as indicated by a significant decrease in activity at later time points for Low and High groups compared with Veh (Figs. 4*A2*,*B2* and 4-2). The decreased durations for both Low and High l-DOPA suggest that changes in the activity of retraction neurons may be important for intermediate and bite BMPs, whereas changes in protraction neurons seemed to be important only for bite BMPs.

10.1523/ENEURO.0206-17.2017.f4-2 Figure 4-2 Statistical comparisons among retraction neurons for Fig. 4. The organization of the table is the same as Fig. 4-1. Kruskal–Wallis test indicated a significant difference between the groups (χ^2^ = 1575.30, *p* = 4.4 × 10^−280^)^i^. Download Figure 4-2, DOC file.

We next examined the effects of l-DOPA on neurons that project axons through specific nerves. Neurons projecting through Rn mediate closure of the radula to grip food (Morton and Chiel, 1993b), whereas neurons projecting through n2 and to a lesser extent n3 mediate backward movement of the radula (Church and Lloyd, 1994). Neurons were separated according to whether they projected axons through Rn, n2, or n3, which was determined by the coincidence of spikes in the neuron with spikes in the nerve (see Fig. 2*E*). We detected only a few neurons with an axonal projection through n1, so this group was excluded. The lack of projections detected in n1 may be because neurons with axons projecting through n1 are primarily located on the other side (rostral) of the ganglion (e.g., B52), are in a deeper cross section (e.g., B67), or originate from another ganglion (e.g., the metacerebral cell located in the cerebral ganglion; Weiss and Kupfermann, 1976). Rn, n2, and n3 projecting neurons were active primarily during the retraction phase (Fig. 4*A3–A5*,*B3–B5*). The activity of Rn projecting neurons persisted for longer in the Veh group compared with Low l-DOPA, as indicated by less activity in Low at later time points compared to Veh (Figs. 4*A3*,*B3* and 4-3). Preparations treated with High l-DOPA had an increase in peak spike frequency compared to Low (Fig. 4-3). The increase in intermediate and bite BMPs due to l-DOPA treatment (Fig. 1) indicates that these treatments shifted the Rn activity from protraction to retraction. Therefore, we predicted that Rn projecting neurons would also shift from protraction to retraction. Surprisingly, the Rn projecting neurons recorded in these experiments were primarily active during retraction in all treatment groups, indicating that l-DOPA treatment did not shift the activity of these neurons from protraction to retraction. These data indicate that Rn neurons in Low l-DOPA had a reduced duration of activity, whereas the High treated group had a boost in frequency and a reduction in duration. n2 projecting neurons had a decrease in duration of activity and increase in spike frequency in High l-DOPA but did not seem to be greatly affected by Low l-DOPA (Figs. 4*A4*,*B4* and 4-4). For n3 projecting neurons, Low l-DOPA treatment led to a reduced duration of activity without changing the peak frequency (Figs. 4*A5*,*B5* and 4-4). However, the peak frequency of n3 neurons was increased in High preparations compared with Low (Fig. 4-5). The increase in frequency of n2 and n3 projecting neurons in High l-DOPA may cause downstream activation of the Rn projecting neurons, as well as activating other neurons, such as B8, that were outside the focal plane. These effects in concert may drive a switch to predominantly bite BMPs. None of these effects among the treatment groups were associated with any differences in the number of neurons classified as protraction (χ^2^ = 0.998, *p* = 0.607; Veh = 0.9 ± 0.3; Low = 0.9 ± 0.3; High = 0.6 ± 0.2),^m^ retraction (χ^2^ = 0.800, *p* = 0.670; Veh = 4.7 ± 1.0; Low = 5.7 ± 0.6; High = 5.4 ± 0.3),^n^ Rn projecting (χ^2^ = 0.236, *p* = 0.889; Veh = 4.6 ± 2.3; Low = 4.0 ± 1.3; High = 2.6 ± 0.5),^o^ n2 projecting (χ^2^ = 1.051, *p* = 0.591; Veh = 9.1 ± 4.7; Low = 12.6 ± 4.2; High = 7.3 ± 2.0),*^p^* or n3 projecting (χ^2^ = 1.628, *p* = 0.443; Veh = 11.9 ± 4.3; Low = 17.6 ± 5.2; High = 7.4 ± 2.0).^q^

10.1523/ENEURO.0206-17.2017.f4-3 Figure 4-3 Statistical comparisons among protraction neurons for Fig. 4. The organization of the table is the same as Fig. 4-1. Kruskal–Wallis test indicated a significant difference between the groups (χ^2^ = 660.15, *p* = 6.3 × 10^−95^)^j^. Download Figure 4-3, DOC file.

10.1523/ENEURO.0206-17.2017.f4-4 Figure 4-4 Statistical comparisons among n2 neurons for Fig. 4. The organization of the table is the same as Fig. 4-1. Kruskal–Wallis test indicated a significant difference between the groups (χ^2^ = 913.19, *p* = 8.1 × 10^−145^)^k^. Download Figure 4-4, DOC file.

10.1523/ENEURO.0206-17.2017.f4-5 Figure 4-5 Statistical comparisons among n3 neurons for Fig. 4. The organization of the table is the same as Fig. 4-1. Kruskal–Wallis test indicated a significant difference between the groups (χ^2^ = 707.03, *p* = 4.9 × 10^−104^)^l^. Download Figure 4-5, DOC file.

The changes observed in peri-event histograms of l-DOPA–treated preparations indicate that specific features of neuronal activity are modulated in a variety of ways to select for intermediate or bite BMPs. Some of these changes could be mediated by shifting the time at which bursts of activity occur in each neuron or involve changes in burst duration and spike frequency within bursts. To gain a better understanding of the ways in which l-DOPA treatment modified activity, we next examined the modulation of burst properties in each of these groups of neurons by l-DOPA treatment.

### l-DOPA uniquely modifies the burst activity of specific subgroups of neurons

Changes in the timing of burst activity can cause dramatic changes to the characteristics of BMPs (e.g., Jing and Weiss, 2001). The timing of burst activity (latency and duration) and activity within bursts (number of spikes and spike frequency; Fig. 5*A*) were measured for every bursting neuron in each BMP, allowing examination of how these features are modified by l-DOPA treatment to switch to intermediate and bite BMPs. For example, a decrease in burst duration and latency combined with an increase in frequency would indicate a compression of spike activity. On the other hand, a reduction in duration without any change in frequency or latency would indicate truncation of spike activity. For the neuronal subgroups defined above, the timing of burst activity is shown visually in Fig. 5*B* and quantitatively in Fig. 5*C*. Results of the statistical analyses are provided in Fig. 5-1. We delineated the major changes induced by l-DOPA treatment.

10.1523/ENEURO.0206-17.2017.f5-1 Figure 5-1 Table summarizing the statistical comparisons for Fig. 5. The neuron subgroup indicated in the first column and the treatment groups being compared are in the second column: LV represents Low vs. Veh, LH represents Low vs. High, VH represents Veh vs. High. A separate Kruskal–Wallis (four total) done on each variable is indicated in the first row. The first row of each comparison is the rank-sum *z* statistic. The second row for each comparison is the Bonferroni-adjusted *p* value. *p* values were capped at one. *, *p* < 0.05, **, *p* < 0.01, and ***, *p*<0.001. Download Figure 5-1, DOC file.

Protraction neurons were modified significantly only by High l-DOPA treatment. In this treatment group, the burst latency was reduced compared with Low l-DOPA without any change in burst duration for either concentration. In addition, protraction neurons had a significant increase in spike frequency within bursts. These results indicate that the bursts in protraction neurons were shifted to an earlier time in relation to the phase with a concomitant boost in spike activity. For retraction, Rn, and n3 neurons, Low l-DOPA treatment did not change the burst latency or the spike frequency but significantly decreased the burst duration and the number of spikes within bursts, indicating that the spike activity of these neurons was truncated (i.e., blocked at later time points without affecting earlier time points) by Low l-DOPA treatment. For Rn, n2, and n3 neurons, High l-DOPA treatment significantly reduced the burst latency and duration and increased the spike frequency within bursts, suggesting that the spike activity was compressed. For n2 neurons, Low l-DOPA treatment significantly decreased the burst duration and increased the spike frequency without changing the burst latency. It is interesting that despite no apparent change in the activity histogram for n2 neurons in Low l-DOPA (Fig. 5*B*,*C*), analysis of individual bursts indicates that changes in n2 were in fact occurring. Retraction neurons treated with High l-DOPA had a decrease in burst latency and duration and in the number of spikes, without a change in spike frequency. These data indicate that Low and High l-DOPA treatments modulate the timing of burst activity for different groups of neurons in different ways. Protraction neurons are most affected by High l-DOPA treatment, whereas retraction, Rn, n2, and n3 projecting neurons are affected by both Low and High l-DOPA treatment, with Low l-DOPA treatment mainly truncating activity and High l-DOPA treatment mainly compressing activity.

### l-DOPA preferentially activates neurons located in different regions of the ganglia

Previous studies in *Aplysia* using backfill tracing have found distinct clusters of neurons projecting through individual nerves (Morton et al., 1991; Scott et al., 1991; Martínez-Rubio et al., 2009; Jelescu et al., 2013); however, backfill tracing cannot examine the distribution of neurons active at particular time points during a BMP. To examine the spatial organization of neurons using VSD imaging of the feeding circuit, we aligned VSD images (see Methods), marked the location of bursting neurons, and pooled the data from all experiments. Analysis of the spatial distribution indicated that protraction, retraction, Rn, n2, and n3 projecting neurons were localized in distinct but overlapping regions of the ganglia (Fig. 6). The distribution of Rn, n2, and n3 projecting neurons roughly agrees with previous observations (Morton et al., 1991; Scott et al., 1991; Jelescu et al., 2013).

We next compared the spatial distribution of the ensemble of neurons active during BMPs in the pooled data of each treatment group (Fig. 7; Video 3). The locations of neurons were marked if the neuron was active with at least one spike during the 0.5-s time bins. Each panel in Fig. 7 includes all the active neurons in all experiments of the indicated treatment group. The spatial distribution of active neurons varied greatly between each time bin and for each treatment. Active neurons tended to be clustered in Low l-DOPA (for example, Fig. 7*A3e*), whereas in the Veh or High l-DOPA groups, the neurons were more widely distributed (e.g., Fig. 7*A4b*). The centroid and pairwise distances of the pooled data were used to quantitatively compare the distribution of active neurons among treatment groups. A line that tracked the centroid of all active neurons across experiments was plotted over time (Fig. 7*B*). The centroid of the Veh group tended to be near the center of the ganglia, whereas the centroids of the Low and High l-DOPA groups tended to be localized more to the upper right (ventrolateral) or left (ventromedial), respectively. The examples in Fig. 7*A2–A4* indicate that the recruitment of many neurons underlies the shift in the centroid. For example, the distributions of activated neurons in panel *e* of Fig. 7*A2–A4* are remarkably different. This result indicates that neurons active in the different treatment groups tended to be localized in different regions of the ganglia.

Video 3. l-DOPA recruits neurons with different spatial distributions. ***A–C*,** Locations of all neurons from all experiments with a given treatment activated at the time bins indicated in ***D***. Each frame represents 0.5 s. The pixels for each overlaying ROI were summed. The number at the top left is the number of neurons activated. The red circle is the centroid of the neurons in the current time bin and the red line is the path of the centroid from previous time bins. Scale bar is 100 μm. ***D***, Timeline of a BMP. 10.1523/ENEURO.0206-17.2017.video.3

The width of the spatial distribution was measured by the average pairwise distance between neurons for each time bin. For all treatment groups, the average pairwise distance increased shortly after the start of the retraction phase. The average pairwise distance was reduced in the Low group compared with High group (χ^2^ = 12.192, *p =* 0.0023; *Q* = –3.451, *p* = 0.0016)^v^ and trended toward a reduction in Low compared with Veh (*Q* = –1.263, *p* = 0.073), indicating that the activated neurons were more tightly clustered in the Low l-DOPA group. The effects of l-DOPA treatment could be caused by variability of the centroid within each treatment group. To examine this possibility, we calculated the centroid for all neurons recruited during protraction or retraction for each preparation. Then, we calculated the distance of these centroids to the mean centroid. The average distance from this mean centroid ranged from 114 to 182 μm (an entire ganglion is ∼1.3 × 0.9 mm), indicating that positions of the neurons were relatively consistent among ganglia during the protraction and retraction phases. Taken together, these results indicate that l-DOPA–induced selection of intermediate and bite BMPs recruited groups of neurons that tended to be located in different but overlapping regions of the ganglia.

## Discussion

**Figure 8.**
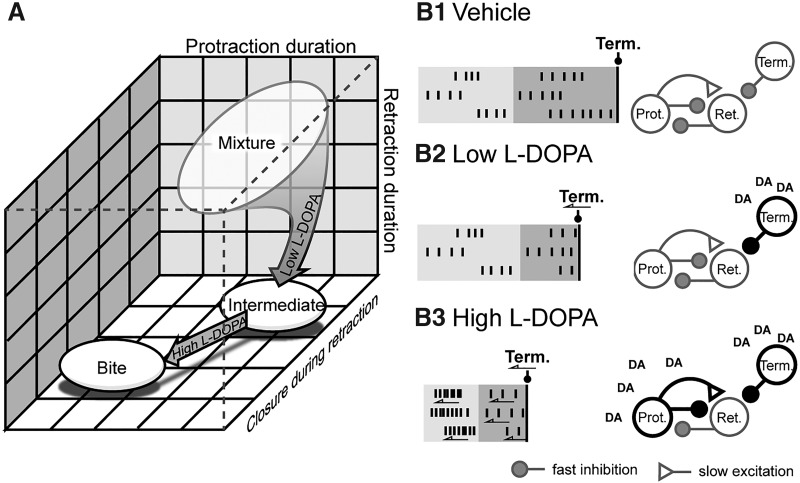
Summary of the l-DOPA effects on neuronal activity. ***A*,** Low l-DOPA biased the selection of BMPs toward intermediates starting from primarily rejection and to a lesser extent swallows (see Veh in Fig. 1*E*). High l-DOPA biased the selection of BMPs toward bite BMPs. ***B*,** Summary of the changes in activity (left) and the proposed mechanism (right). ***B1*,** Activity in Veh. Vertical lines signify action potentials. Light gray is protraction phase. Dark gray is retraction phase. Black line is the onset of activity of BMP terminating (Term.) neurons. Gray outlines indicate basal conditions; black outlines indicate enhancement. Protraction spike frequency is enhanced. ***B2*,** Activity in Low l-DOPA. The burst are truncated because the terminating neurons are being activated sooner (arrow). ***B3*,** Activity in High l-DOPA. The enhancement of protraction neurons more rapidly activates retraction neurons.

Combined VSD and nerve recordings of isolated buccal ganglia revealed that Low l-DOPA biased the feeding network toward intermediate BMPs and High l-DOPA biased the network toward bite BMPs, whereas Veh seemed to express a mixture of BMPs (Fig. 8*A*). Previous studies in semi-intact *Aplysia* preparations indicate that l-DOPA and DA treatment modulate feeding behavior (Kabotyanski et al., 2000). Therefore, low and high levels of dopamine may contribute to the expression of intermediate and bite behaviors respectively *in vivo*. Although intermediate behaviors have been observed *in vivo* (Morton and Chiel, 1993a,b), their function remains unclear. Intermediate behaviors may serve to reposition or cut food in the mouth cavity (Hurwitz and Susswein, 1992; Morton and Chiel, 1993a).

Similar to the results obtained by Kabotyanski et al. (2000), the effects of l-DOPA treatment occurred within 15 min. l-DOPA is likely enhancing the release of DA from dopaminergic neurons within the buccal ganglia (e.g., B65 and B20; Kabotyanski et al., 1998; Jing and Weiss, 2001). However, increased spontaneous release throughout the ganglia from dopaminergic afferents from the esophageal nerve is another possibility. Given these two possibilities, it is unclear whether the bias in motor patterns by l-DOPA treatment is due to increased DA acting directly on a large number of neurons, via the esophageal nerve, or due to DA action on a smaller number of highly connected neurons, via B65 and B20. If the effects of l-DOPA treatment are due to enhanced release from B65 and B20, then these effects are dependent on the dynamics of the activity of these neurons. Low and High l-DOPA treatment induced a wide variety of effects on neuronal activity (Figs. 4-5). The differential modulation of neurons suggests that l-DOPA treatment is not simply enhancing or suppressing activity overall. Currently, it is unclear what molecular mechanisms mediate this differential modulation. In mammals, differential modulation is mediated at least in part by the selective expression of D1-like and D2-like receptors, which have different sensitivities to dopamine (Beaulieu and Gainetdinov, 2011). A D1-like receptor has been characterized in *Aplysia* (Barbas et al., 2006), and some of the components of its downstream signaling cascade are important for the modulatory effects of DA on the identified retraction neuron B51 (Lorenzetti et al., 2008). A genome-wide sequencing has also predicted a D2-like receptor (NCBI: NW_004797500.1). An intriguing possibility is that neurons express different ratios of D1 and D2-like receptors, similar to the striatum in mammals (Schultz, 2013). Low l-DOPA treatment could predominantly activate high-affinity D2 receptors, resulting in a switch to intermediate BMPs, whereas High l-DOPA treatment could predominantly activate low-affinity D1 receptors, resulting in a switch to bite BMPs.

High l-DOPA treatment led to an increase in the number of BMPs (Fig. 3) and an increase in protraction neuron activity (Figs. 4*A*, 5*B*). This increase could be due to an increase in excitability of protraction neurons. Indeed, increasing the excitability of protraction neurons (i.e., B30, B63, and B65) enhances the frequency of BMPs (Sieling et al., 2014). In addition, DA increases the excitability and spike frequency of a protraction phase neuron, B67 (Serrano and Miller, 2006). Increased excitability would cause these neurons to be activated more rapidly, decreasing the burst latency (Fig. 8*B3*). The enhanced activation of protraction neurons could, in turn, increase synaptic drive through putative excitatory synaptic connections to more rapidly activate retraction, Rn, n2, and n3 neurons, which terminate the protraction phase by feedback inhibition (e.g., Hurwitz and Susswein, 1996). BMPs are terminated by inhibitory neurons (e.g., B52) activated via rebound excitation at the end of the retraction phase (Fig. 8*B1*; Plummer and Kirk, 1990). DA also increases the rebound excitation and sag potential in neuron B8 (Kabotyanski et al., 1998; Díaz-Ríos and Miller, 2005). One intriguing possibility is that Low and High l-DOPA treatments increase the rebound excitation or sag potential of BMP-terminating neurons such as B52, causing these neurons to activate earlier, and in turn terminating the activity of retraction neurons (Fig. 8*B2–3*). Kabotyanski et al. (2000) found that application of 250 μM (High) l-DOPA decreased the excitability of B4, B34, and B64. The decrease in B4 and B34 would bias the output toward bite motor patterns. The decrease in B64 excitability is consistent with the decrease in retraction duration. In addition, High l-DOPA enhanced the strength of the B64-to-B31/32 inhibitory synaptic connection (Kabotyanski et al., 2000). B64 is a retraction neuron and B31/32 is a protraction neuron; therefore, increasing the strength of this feedback inhibitory connection is consistent with the result that High l-DOPA treatment decreases the protraction phase. Finalely, High l-DOPA decreased the strength of the B64-to-B4 excitatory synaptic connection, which would bias the motor output toward bite motor patterns (Kabotyanski et al., 2000). The observations provided by VSD experiments combined with the results from intracellular studies provide a detailed picture of the mechanisms underlying the change in motor patterns induced by l-DOPA.

The Rn projecting neuron B8 shifts from being primarily active during protraction in rejection BMPs to being primarily active during retraction in bite and swallow BMPs (Morton and Chiel, 1993b). Unexpectedly, we did not observe a shift in the phase of activity in Rn projecting neurons as we did for the Rn activity. B8 is typically below the focal plane of our recordings. Thus, an explanation for this discrepancy is that the Rn projecting neurons we recorded were not likely to be B8 and thus were not likely responsible for large-unit activity in Rn.

We also found that neurons with different properties (i.e., preferred phase of activity or axonal projections) are located in distinct but overlapping regions of the ganglia (Fig. 7). Neurons with similar properties or functions may be connected with chemical or electrical synapses, which may be facilitated by such topographical organization. For example, B31 and B32 as well as B4 and B5 are highly coupled to each other and are adjacent to each other (Gardner, 1977; Susswein and Byrne, 1988). In addition, different concentrations of l-DOPA recruited ensembles of neurons with different spatial distributions. In the future, more comprehensive analytic methods could be used to identify or match the correspondence of neurons across preparations, as has been done for the leech locomotor CPG (Kapoor et al., 2015; Frady et al., 2016). Identification of specific neurons would allow for conventional electrophysiological methods to examine the underlying biophysical mechanisms of the observed changes in activity.

We observed a general increase in rhythmic motor patterns and alterations to motor patterns depending on the level of l-DOPA and presumably enhancement of DA release by l-DOPA, which is similar to studies in other systems. In leeches, DA application elicits rhythmic bursts of motor neurons in a crawl-like pattern (Puhl and Mesce, 2008; Puhl et al., 2012), and DA increases the rebound excitation and decreases the AHP to different extents in two crawl-related motor neurons (Crisp et al., 2012). In lampreys, DA affects swim patterns in a concentration-dependent manner, with low concentrations (0.1–10 µM) increasing swim frequency and higher concentrations decreasing swim behavior. The increase in swim is due to an increase in the excitability (via suppression of AHP) of motor neurons, edge cells, giant interneurons, and dorsal cells, as well as a decrease in inhibition from commissural interneurons (Kemnitz, 1997). DA evokes multirhythmic motor patterns in neonatal mice (Sharples and Whelan, 2017) and increases the excitability of motor neurons and the glutamatergic transmission they receive (Han et al., 2007). In the above studies, a common theme is an enhancement in excitability due to DA-induced reduction in the AHP. Future investigations could examine whether the increases in activity observed in High l-DOPA in *Aplysia* feeding are likewise mediated by a decrease in the AHP. For example, a reduction of the AHP (in the soma or axon) could increase the activity of protraction neurons. In addition, l-DOPA could lead to modifications of additional ionic currents. For example, in the lobster pyloric network, bath application of DA produced opposite effects on neurons (e.g., enhancement of the I_A_ current in the pyloric dilator neuron and attenuation of this current in the anterior burster neuron; Harris-Warrick et al., 1998).

In conclusion, the results from this study indicate that different levels of DA enhancement modulate neurons in distinct ways to bias the feeding circuit toward specific motor patterns. Additional analysis revealed characteristic alterations in the burst properties and spatial distribution of recruited neurons. Understanding DA modulation of the *Aplysia* feeding central pattern generating network may help to improve understanding of DA modulation of more complex networks in the vertebrate CNS.

## Synthesis

Reviewing Editor: Elizabeth Cropper, Mt Sinai Sch Med Decisions are customarily a result of the Reviewing Editor and the peer reviewers coming together and discussing their recommendations until a consensus is reached. When revisions are invited, a fact-based synthesis statement explaining their decision and outlining what is needed to prepare a revision will be listed below. The following reviewer(s) agreed to reveal their identity: Thomas Abrams, Abraham Susswein.

There was a great deal of enthusiasm for this manuscript. The reviewers refer to it as ‘groundbreaking’ ‘unique’, and ‘rigorous’. The general consensus is that no additional experiments are needed, but that several issues should be clarified. Issues of particular importance are summarized below. After that I have included the full list of major and minor comments as received from the reviewers.

**Major points to be clarified**

*Classification of motor program* The reviewers understand the technical limitations of this work, which prompted the use of n1 as a monitor of protraction. However, they would like to see further discussion of the limitations of this approach. Further, they felt that it is important to define ‘large unit activity’ and how it was used to classify programs as rejections or intermediate programs.

*Are intermediate programs physiological?* Both reviewers would like to see further discussion of this issue.

*The use of L-DOPA to evoke motor programs* This point generated the most discussion and confusion. One reviewer felt that the bias of this paper was to suggest that the dopamine that is eventually released is acting globally on many neurons. This reviewer felt that this was an interesting point that differs from what is in the literature. Consequently, they felt that this issue should be explicitly discussed. The other reviewer was less convinced that the intent was to suggest a global effect. This reviewer felt that it is impossible to distinguish from these experiments whether the released DA modulates the buccal pattern by acting focally on pattern generator neurons or acting over a longer distance, more diffusely. Consequently, there was no intent to imply either possibility. To eliminate this sort of confusion both reviewers agreed that there should be further discussion of the presumed source of dopamine and where it acts.

**Full list of reviewers’ comments**

Reviewer 1

This is a unique study that used voltage sensitive dye recordings for a system level analysis of dopamine modulation of the well-understood feeding pattern generator of Aplysia. In an ingenious strategy, the authors used exposure to L-DOPA to increase dopaminergic signaling, either modestly or strongly, depending on the L-DOPA concentration. The results are dramatic and the effects of increased dopaminergic signaling appear very robust. The thorough analysis demonstrating the reliable detection of action potentials using voltage sensitive dye is elegant and makes the study quite rigorous.

None of my comments below represent major concerns. Most relate to wording or data presentation.

The rationale for presenting the position of the centroid within behaviors and across treatment groups is somewhat obscure. The comment “The path of the centroid of the Low never overlapped with the High and only briefly overlapped with Veh” has little significance. If the overall population shifted only a few somata, one could in principle observe such a shift in the centroid. Thus, with the addition or loss of just a few active neurons, the centroid could shift. It is not at all obvious how one would assess whether there was a meaningful change in position of the active neurons, or what constitutes a meaningful change. This analysis is tougher because the different L-DOPA levels were apparently tested in different ganglia. “This result indicates that neurons active in the different treatment groups tended to be localized in different regions of the ganglia.” Is there really evidence to support this conclusion? Also, this presentation appears to make the assumption that there are functionally relevant distinct maps across the buccal ganglion for different motor patterns; however, there does not seem to be a clear analysis of the neurons in Fig. 3A. What would results look like if the full group of active neurons for each specific phase of a motor program were compared across programs?

Were there separate ganglia for each treatment? The Results state each pair of ganglia received a single treatment. This point should be more clearly stated in Methods and in relevant figures, particularly the legend for Fig. 3.

Intermediate Buccal Motor Programs - these are associated with little effective injection or rejection of food. Do these behaviors occur in intact animals, or is this abnormal behavior shown only in the physiology preparations? Put differently, do retraction neurons actually experience low concentrations of dopamine, too low to modulate protraction neurons? This is not to suggest that the authors should perform voltammetry measurements for DA. Instead the authors should address the issue of whether such intermediate motor programs, which might be initiated by low DA concentrations, are common in normal behavior.

In some parts of their analysis, the authors examine shifts in the spatial pattern of activity across the buccal ganglion during the various phases of buccal motor programs. It would be worthwhile to comment on the consistency of the positions of buccal neurons involved in the motor programs, as the authors have a unique ability to view the overall network of neurons.

Including the nerve IDs (Run, n2 and n3) in the Abstract to refer to populations of neurons based on their peripheral projections makes it appear that the manuscript is less accessible than it is; these details are likely to deter non-specialists from reading the paper.

l. 39. “However, little is known about DA modulation of individual neuronal activity of larger networks” seems a bit confusing, and merits clarification. Usually investigations of larger neuronal networks are inherently unable to monitor changes in individual neurons. The authors' unique approach enables bridging of these levels of analysis.

l. 88. Three times normal divalents. If the normal concentration of [Ca] + [Mg] is 60 mM, 3x would be 180 mM. The authors work with a total divalent concentration of 140 mM, which is less than 2.5 times normal.

l. 112. The large unit activity recorded from n1 was used as an indicator of the start of the protraction and retraction phases. Yet n1 was excluded from the analysis because few neurons projected through n1 (l. 425); this requires further explanation.

l. 374. “Low (L-DOPA) somewhat decreased the synchrony ...” What does “~0.1” signify? How were pairwise correlations calculated? This is mentioned minimally in Methods. There should be more explanation in Results if values are presented. Note, “Low” is not followed by a noun.

l. 530. “whereas Veh seemed to express primarily rejections and swallows” It is unclear that the vehicle control promotes any particular form of BMP (as compared with the BMP types that are generated by the naive circuit.

l. 532. The distinction between “dopaminergic afferents” and B65 and B20 would be clearer for those unfamiliar with this system if “buccal ganglion” preceded dopaminergic neurons.

l. 535. The sentence suggesting that dopamine is “specifically modulating neurons in different ways” seems rather trivial. If there is a more interesting point here, it should be stated clearly, or the sentence could be shortened and combined with the next one.

l. 546 and 547. L-DOPA does not activate neurons, but leads, indirectly to increased activation. The same comment applies to “specifically modulating” on l. 536 and “modulation” on l. 453. The language is more precise in the Significance Statement and Introduction.

l. 567 and Results. The lack of a shift in phase of Rn projecting neurons (measured with VSD) was not so clearly presented in Results. The shift in the pattern of Rn activity should be emphasized and contrasted with the VSD results.

l. 579. “These ensembles could be specific to the BMPs (e.g., an intermediate or bite ensemble) or could be specific to the concentrations of L-DOPA.” This dichotomy does not make sense. Clearly, different concentrations of L-DOPA were associated with different patterns of neuronal activity, which are detected as different ensembles of cells. Moreover, the assumption throughout the study has been that neurons that project in different nerves participate in different motor programs. Thus, there do not seem to be mutually exclusive alternatives here and the first is certainly correct and the second also is sup[ported by the data presented throughout the study. The proposed experiments ** are nevertheless interesting, but they don't address the relationship of activity patterns to L-DOPA concentrations.

Number of bursts examined. It would be worthwhile in the legends to indicate the number of animals as well as the number of bursts.

Centroid opacity increases at later time points. Although there is concern about the importance of the centroid analysis, this is interesting. The legend does not make it clear whether the opacity represents a component of the data or simply the start vs. the end of the sequence.

What are the black bars in Figure 1B just below the Swallow, Intermediate and Bite labels? This should be clearly defined in the legend.

For Fig. 2E1, it would be helpful to label the nerve and VSD traces.

Fig. 7. The centroid map in B should indicate more clearly the beginning and end of a burst, perhaps with arrows. Moreover, these maps seem to include approximately 14 time points whereas the data in panel A suggests 5 or 10 time points. This merits explanation. For this analysis, is this for single animals and multiple bursts?

Reviewer 2

This is a groundbreaking paper. It presents a completely new approach to study a reasonably well-studied neural system. This is both its strength and its weaknesses.

The paper uses combined extracellular recordings from nerves and voltage sensitive dye recordings from multiple neurons to characterize changes in motor activity in the buccal ganglia in response to 2 concentrations of the dopamine precursor L-DOPA. The paper reports that a low dosage of L-DOPA biases the motor system to produce intermediate programs, whereas a high does induces bite-like programs. This is an apparent continuation of previous work by Kabotyanski and collaborators on how dopamine affects motor activity, but the newly introduced techniques of combined dye and extracellular recordings allow the present investigators to go well beyond the previous conclusions. They are now able to examine synchrony of activity across populations of neurons, and to determine the means by which a program is speeded up by dopamine. They are also able to examine whether neurons that fire in phase are co-localized in the buccal ganglia. They report that neurons firing in different phases of motor activity are localized to different area of the ganglia.

The data presented are recordings from many unidentified protraction-phase and retraction-phase neurons, and conclusions are drawn from the recordings. These are interesting and important conclusions, but they are divorced from all previous work that explained the buccal ganglion circuitry and its functions in terms of the properties and connections between identified neurons. The conclusions in this paper should be integrated with the previous findings on control of feeding via identified neurons, and the ideas that have been developed on the functions of these neurons. This means softening conclusions and inferences in the current paper, and tying them to what is known from other work on this circuit.

I do not underestimate the work that has gone into this paper. An entirely new set of experimental techniques had to be developed, and new tools to analyze the data had to be used. This is an important paper that **must** be published, but it also must be tied to the previous work.

Specific comments:

Lines 64-65. “We found that a low concentration of L-DOPA biased motor activity toward intermediate BMPs, whereas a high concentration of L-DOPA biased motor activity towards bite BMPs.” What was the rationale of examining different concentrations of L-DOPA? Is there any evidence that dopamine is released in the buccal ganglia as a general modulator of feeding via modulatory effects on many neurons, rather than as a transmitter utilized by a number of distinct dopaminergic neurons? Are the different effects of low and high concentrations physiological mechanisms that are used to control different behaviours in the buccal ganglia? The authors should address themselves to these questions.

Line 78: the authors placed the buccal mass in high divalent cation solution. This is generally used to block polysynaptic connections. Wouldn't it have made more sense to work in a high Mg2+ solution, to block all synaptic transmission?

Line 122 - “retraction” should be “rejection”.

Line 139-140 -“These time points were chosen because this approximated the shape of a typical action potential in Aplysia”. Some very important *Aplysia* buccal ganglia neurones are very atypical.

Line 152 - “ran” should be run.

Lines 244-245 - “The first phase is 245 protraction, defined here as large unit activity in buccal nerve 1 (n1)...” The best defined protraction-phase neurons are B31, B32, B61, B62, which all have axons in the I2 nerve, rather than in n1. The axons in n1 are from B67, which innervates the salivary glands, and probably from unidentified neurons that innervate the most anterior region of the esophagus and its connection to the buccal mass. In my experience, B67 activity begins somewhat before activity in the I2 nerve and muscle, so these recordings are at best a loose correlate of protraction.

Line 250 - “Activity of the radula nerve 1 (Rn) mediates closure movement in vivo.” I would say that it is a correlate of closure. B8 mediates closure and has an axone in the radula nerve, but the nerve per se does not mediate closure.

Lines 283-286 - “Reduction in retraction neuron activity duration due to Low L-DOPA may bias the network towards intermediate BMPs, whereas combined reductions in protraction and retraction neuron activity duration due to High L-DOPA may bias the network towards bite BMPs.” The implication is that this is a group decision, based on the responses of many neurons to dopamine, rather than a small number of neurons that set the pattern. Could be, but the authors should point out how this approach is different from that previously thought to work, in that the activation of specific neurones sculpts the response.

In addition, why would low L_DOPA bias the network to intermediates buccal motor programs? Do such buccal motor programs have any function?

Lines 347-350 - “The VSD recordings revealed that the L-DOPA-induced changes in BMPs as monitored via nerve recordings (Fig. 1) are due to enhanced rhythmic activity in a large number of neurons in the buccal ganglion.” I would take out “due to” and put in “correlated with”. The neurones recorded by VSD may not be those recorded in the extracellular trace.

Lines 392-393 - “We first examined protraction neurons, which have many important functions such as initiating BMPs and protracting the radula outward to grasp food.” But you could not have recorded from the neurones that have these functions, since they are somewhat deeper than the surface and some have truncated, very small action potentials. The sentence should actually read: “We first examined protraction phase neurons of unknown function, whose firing is correlated with neurons which have many important functions such as initiating BMPs and protracting the radula outward to grasp food.”

Lines 414-417 - “The decreased durations for both Low and High L-DOPA suggest that changes in the activity of retraction neurons may be important for switching to both intermediate and bite BMPs, whereas changes in protraction neurons seemed to be important only for switching to bite BMPs.” What is the function of switching to intermediate buccal motor programs? Again, is choice of a behavior mediated by regulating the activity of many cells by a specific concentration of transmitter, rather than by recruiting one or a number of neurons, such as B20, B34, B40, or B51? It may be, but the authors must make explicit that they are proposing a new mechanism of behavioural choice that differs from those previously suggested in this system.

Lines 531-534 - “L-DOPA is likely enhancing the release of DA from dopaminergic neurons B65 and B20 (Kabotyanski et al., 1998; Jing and Weiss, 2001). However, increased spontaneous release from dopaminergic afferents is another possibility.” Is there any evidence that dopaminergic neurones dynamically and quickly respond to L-DOPA?

Line 552 - Seiling et al., 2014 - Not in the reference list. In addition, I could not find it in PubMed or in the Society for Neuroscience Abstracts for 2014.

Line 553 - protraction neuron B67 - B67 is NOT a protraction neurone. It is a protraction-**phase** neurone. There are no data on how DA affects the cells that actually produce protraction.

Lines 555-556 - “ The enhanced activation of protraction neurons could, in turn, increase synaptic drive to more rapidly activate retraction...” Retraction is initiated by B64. There are no known strong connections between the protraction neurons and B64.

Lines 600-604 - speculate that DA could act primarily by regulating AHP. But the real protractors, B31 and B32, show no AHP after each spike, since the spikes are superimposed on a 30-40 mV sustained plateau potential, and in any case do not invade the soma.

Line 782 - “large unit activity” Please define large unit activity. This is not a trivial point. Figure 1B2 shows Rn activity in both protraction and retraction, but the units active in protraction are much larger than are those active in retraction. Nonetheless, both are being classified as “large unit activity”, since these are being classified as intermediate programs. If only the larger spikes were counted, these programs would be rejection-like (I would have classified them as rejections, based on the size of the Rn spikes during protraction, and the prominent n3 activity at the start of retraction, which probably is indicative of B4/B5 activity).

Lines 800-801 - A1, A2: The text (lines 293-294) says that these recordings are from B4, but the Legend does not mention this.

Lines 852-859 - I do not understand the figure or the Legend. Please explain this more clearly.

Line 890 - “L-DOPA recruits neurons with different spatial distributions.” So these are not the same neurons. So some sort of global regulation of a homogeneous population of neurons by DA could not account for the data. But much of this paper certainly suggests that DA works this way.

Extended Data Legends. The Tables are impossible to understand, since they are unlabelled. The explanation in the legend is not adequate to understand the tables. Please put labels into the Tables.
