# Supplementary material for: Unique Configurations of Compression and Truncation of Neuronal Activity Underlie l-DOPA–Induced Selection of Motor Patterns in Aplysia
Source: eNeuro. 2017 Oct 24;4(5):ENEURO.0206-17.2017. doi: 10.1523/ENEURO.0206-17.2017 (PMC5654236; doi:10.1523/ENEURO.0206-17.2017)
Supplement: Figure 4-1 [file enu005172435so13.doc]

| Time  bin(s) | Low vs Veh | | Low vs High | | Veh vs High | |
| --- | --- | --- | --- | --- | --- | --- |
|  | *t*-value | P-value | *t*-value | P-value | *t*-value | P-value |
| -6.0 | 1.41 | 1 | 0.56 | 1 | -1.09 | 1 |
| -5.5 | 1.48 | 1 | 3.39 | 0.053 | 1.09 | 1 |
| -5.0 | 0.4 | 1 | 3.92 | **0.0068 | 2.69 | 0.54 |
| -4.5 | 1.85 | 1 | 5.43 | ***4.2x10^-6^ | 2.31 | 1 |
| -4.0 | 1.17 | 1 | 4.09 | **0.0032 | 1.99 | 1 |
| -3.5 | 1.97 | 1 | 5.15 | ***2.0x10^-5^ | 1.96 | 1 |
| -3.0 | 0.99 | 1 | 4.63 | ***2.7x10^-4^ | 2.62 | 0.67 |
| -2.5 | 2.42 | 1 | 6.4 | ***1.2x10^-8^ | 2.47 | 1 |
| -2.0 | 1.8 | 1 | 3.8 | *0.011 | 1.06 | 1 |
| -1.5 | 3.51 | *0.034 | 0.03 | 1 | -3.81 | **0.01 |
| -1.0 | 1.76 | 1 | -3.57 | *0.027 | -4.78 | ***1.3x10^-4^ |
| -0.5 | 2.22 | 1 | -3.74 | *0.014 | -5.42 | ***4.5x10^-6^ |
| 0.0 | 0.5 | 1 | -2.99 | 0.21 | -2.94 | 0.25 |
| 0.5 | 1.46 | 1 | 2.4 | 1 | 0.32 | 1 |
| 1.0 | 0.87 | 1 | 1.66 | 1 | 0.38 | 1 |
| 1.5 | 0.43 | 1 | 1.06 | 1 | 0.38 | 1 |
| 2.0 | -0.19 | 1 | 0.11 | 1 | 0.3 | 1 |
| 2.5 | 0.19 | 1 | 0.1 | 1 | -0.13 | 1 |
| 3.0 | 0.06 | 1 | 0.03 | 1 | -0.04 | 1 |
| 3.5 | 0.19 | 1 | -0.28 | 1 | -0.43 | 1 |
| 4.0 | 0.96 | 1 | -0.01 | 1 | -1.06 | 1 |
| 4.5 | 0.59 | 1 | -3.12 | 0.13 | -3.14 | 0.13 |
| 5.0 | 1.36 | 1 | -3.63 | *0.021 | -4.39 | ***8.4x10^-4^ |
| 5.5 | 0.91 | 1 | -5.26 | ***1.1x10^-5^ | -5.2 | ***1.5x10^-5^ |
| 6.0 | 2.19 | 1 | -1.04 | 1 | -3.23 | *0.093 |
