# Supplementary material for: Unique Configurations of Compression and Truncation of Neuronal Activity Underlie l-DOPA–Induced Selection of Motor Patterns in Aplysia
Source: eNeuro. 2017 Oct 24;4(5):ENEURO.0206-17.2017. doi: 10.1523/ENEURO.0206-17.2017 (PMC5654236; doi:10.1523/ENEURO.0206-17.2017)
Supplement: Figure 4-2 [file enu005172435so14.doc]

| Time  bin(s) | Low vs Veh | | Low vs High | | Veh vs High | |
| --- | --- | --- | --- | --- | --- | --- |
|  | *t*-value | P-value | *t*-value | P-value | *t*-value | P-value |
| -6.0 | 1.68 | 1 | -3.45 | *0.042 | -4.37 | ***9.4x10^-4^ |
| -5.5 | 0.71 | 1 | -5.96 | ***1.8x10^-7^ | -5.18 | ***1.7x10^-5^ |
| -5.0 | 0.66 | 1 | -6.51 | ***5.8x10^-9^ | -5.53 | ***2.5x10^-6^ |
| -4.5 | 0.69 | 1 | -7.17 | ***5.5x10^-11^ | -6.05 | ***1.1x10^-7^ |
| -4.0 | 0.84 | 1 | -3.6 | *0.027 | -3.57 | *0.027 |
| -3.5 | 1.79 | 1 | -0.46 | 1 | -2.28 | 1 |
| -3.0 | 0.65 | 1 | -0.2 | 1 | -0.86 | 1 |
| -2.5 | 0.8 | 1 | 1.24 | 1 | 0.04 | 1 |
| -2.0 | 0.18 | 1 | 1.76 | 1 | 1.11 | 1 |
| -1.5 | -0.53 | 1 | 0.1 | 1 | 0.64 | 1 |
| -1.0 | 0.02 | 1 | 0.68 | 1 | 0.48 | 1 |
| -0.5 | 0.39 | 1 | 0.49 | 1 | -0.06 | 1 |
| 0.0 | 2.24 | 1 | -1.53 | *0.027 | -3.57 | *0.027 |
| 0.5 | 0.83 | 1 | -2.77 | 0.24 | -2.95 | 0.24 |
| 1.0 | 0.02 | 1 | -2.14 | 1 | -1.6 | 1 |
| 1.5 | 1.46 | 1 | 2.03 | 1 | -0.08 | 1 |
| 2.0 | -0.69 | 1 | 3.19 | 0.14 | 3.1 | 0.14 |
| 2.5 | -2.36 | 1 | 2.32 | **0.0014 | 4.28 | **0.0014 |
| 3.0 | -2.93 | 0.25 | 1.57 | *0.0011 | 4.34 | *0.0011 |
| 3.5 | -3.93 | **0.0063 | -0.88 | *0.022 | 3.62 | *0.022 |
| 4.0 | -2.89 | 0.29 | 1.66 | ***9.5x10^-4^ | 4.37 | ***9.5x10^-4^ |
| 4.5 | -2.13 | 1 | 1.2 | 0.10 | 3.2 | 0.10 |
| 5.0 | -1.98 | 1 | -0.14 | 1 | 2.05 | 1 |
| 5.5 | -1.36 | 1 | -1.99 | 1 | 0 | 1 |
| 6.0 | -0.83 | 1 | -3.79 | 1 | -1.91 | 1 |
