# Supplementary material for: Unique Configurations of Compression and Truncation of Neuronal Activity Underlie l-DOPA–Induced Selection of Motor Patterns in Aplysia
Source: eNeuro. 2017 Oct 24;4(5):ENEURO.0206-17.2017. doi: 10.1523/ENEURO.0206-17.2017 (PMC5654236; doi:10.1523/ENEURO.0206-17.2017)
Supplement: Figure 4-3 [file enu005172435so15.doc]

| Time  bin(s) | Low vs Veh | | Low vs High | | Veh vs High | |
| --- | --- | --- | --- | --- | --- | --- |
|  | *t*-value | P-value | *t*-value | P-value | *t*-value | P-value |
| -6.0 | 0.35 | 1 | -3.67 | *0.018 | -2.52 | 0.87 |
| -5.5 | 0.21 | 1 | -4.29 | *0.0014 | -2.74 | 0.46 |
| -5.0 | -0.23 | 1 | -4.29 | **0.0013 | -2.3 | 1 |
| -4.5 | -0.12 | 1 | -4.42 | ***7.3x10^-4^ | -2.5 | 0.94 |
| -4.0 | 0.75 | 1 | -1.62 | 1 | -1.72 | 1 |
| -3.5 | 1 | 1 | -0.69 | 1 | -1.42 | 1 |
| -3.0 | 0.01 | 1 | 0.07 | 1 | 0.04 | 1 |
| -2.5 | 0.08 | 1 | 0.53 | 1 | 0.24 | 1 |
| -2.0 | -0.57 | 1 | 1.48 | 1 | 1.45 | 1 |
| -1.5 | -0.65 | 1 | 0.69 | 1 | 1.07 | 1 |
| -1.0 | -0.14 | 1 | 0.61 | 1 | 0.5 | 1 |
| -0.5 | -0.76 | 1 | 1.36 | 1 | 1.57 | 1 |
| 0.0 | 0.52 | 1 | 1.94 | 1 | 0.62 | 1 |
| 0.5 | -2.08 | 1 | -3.03 | 0.18 | 0.31 | 1 |
| 1.0 | -1.41 | 1 | -5.03 | *3.8x10^-5^ | -1.54 | 1 |
| 1.5 | -1.29 | 1 | -0.36 | 1 | 1.09 | 1 |
| 2.0 | -1.72 | 1 | 2.13 | 1 | 3 | 0.20 |
| 2.5 | -3.3 | 0.071 | -2.22 | 1 | 2.04 | 1 |
| 3.0 | -3.69 | *0.017 | -3.47 | *0.039 | 1.69 | 1 |
| 3.5 | -4.21 | **0.0019 | -5.09 | ***2.6x10^-5^ | 1.26 | 1 |
| 4.0 | -2.27 | 1 | -0.29 | 1 | 2.13 | 1 |
| 4.5 | -2.01 | 1 | 0.08 | 1 | 2.08 | 1 |
| 5.0 | -1.86 | 1 | -1.15 | 1 | 1.21 | 1 |
| 5.5 | -2.44 | 1 | -1.3 | 1 | 1.17 | 1 |
| 6.0 | -1.89 | 1 | -1.07 | 1 | 1.29 | 1 |
