# Supplementary material for: Unique Configurations of Compression and Truncation of Neuronal Activity Underlie l-DOPA–Induced Selection of Motor Patterns in Aplysia
Source: eNeuro. 2017 Oct 24;4(5):ENEURO.0206-17.2017. doi: 10.1523/ENEURO.0206-17.2017 (PMC5654236; doi:10.1523/ENEURO.0206-17.2017)
Supplement: Figure 4-4 [file enu005172435so16.doc]

| Time  bin(s) | Low vs Veh | | Low vs High | | Veh vs High | |
| --- | --- | --- | --- | --- | --- | --- |
|  | *t*-value | P-value | *t*-value | P-value | *t*-value | P-value |
| -6.0 | 0.3 | 1 | -3.31 | 0.070 | -2.67 | 0.58 |
| -5.5 | 0.07 | 1 | -4.46 | ***6.2x10^-4^ | -3.23 | 0.093 |
| -5.0 | -1.03 | 1 | -4.99 | ***4.5x10^-5^ | -2.44 | 1 |
| -4.5 | -0.26 | 1 | -4.85 | ***9.3x10^-5^ | -3.15 | 0.12 |
| -4.0 | -0.2 | 1 | -2.61 | 0.69 | -1.63 | 1 |
| -3.5 | 0.41 | 1 | -0.99 | 1 | -1.14 | 1 |
| -3.0 | 0.05 | 1 | -0.26 | 1 | -0.24 | 1 |
| -2.5 | 0.22 | 1 | 0.29 | 1 | -0.03 | 1 |
| -2.0 | -0.47 | 1 | -0.29 | 1 | 0.3 | 1 |
| -1.5 | 0.3 | 1 | -1.39 | 1 | -1.3 | 1 |
| -1.0 | -0.64 | 1 | -2.53 | 0.86 | -1.11 | 1 |
| -0.5 | 0.23 | 1 | -2.32 | 1 | -1.89 | 1 |
| 0.0 | 0.33 | 1 | -0.66 | 1 | -0.81 | 1 |
| 0.5 | -0.16 | 1 | -3.93 | **0.0063 | -2.61 | 0.68 |
| 1.0 | -1.53 | 1 | -4.73 | *1.7x10^-4^ | -1.72 | 1 |
| 1.5 | -1.19 | 1 | 0.99 | 1 | 1.96 | 1 |
| 2.0 | -3.78 | *0.012 | 2.03 | 1 | 5.47 | ***3.4x10^-6^ |
| 2.5 | -4.84 | ***9.5x10^-5^ | -1.15 | 1 | 4.35 | ***0.0010 |
| 3.0 | -4.08 | *0.0034 | -2.16 | 1 | 2.82 | 0.36 |
| 3.5 | -3.18 | 0.11 | -2.35 | 1 | 1.73 | 1 |
| 4.0 | -1.7 | 1 | 0.21 | 1 | 1.96 | 1 |
| 4.5 | -1.41 | 1 | 0.27 | 1 | 1.69 | 1 |
| 5.0 | -1.17 | 1 | -0.32 | 1 | 1.02 | 1 |
| 5.5 | -1.68 | 1 | -1.98 | 1 | 0.39 | 1 |
| 6.0 | -0.75 | 1 | -5.15 | ***2.0x10^-5^ | -2.84 | 0.34 |
