# Supplementary material for: Unique Configurations of Compression and Truncation of Neuronal Activity Underlie l-DOPA–Induced Selection of Motor Patterns in Aplysia
Source: eNeuro. 2017 Oct 24;4(5):ENEURO.0206-17.2017. doi: 10.1523/ENEURO.0206-17.2017 (PMC5654236; doi:10.1523/ENEURO.0206-17.2017)
Supplement: Figure 4-5 [file enu005172435so17.doc]

| Time  bin(s) | Low vs Veh | | Low vs High | | Veh vs High | |
| --- | --- | --- | --- | --- | --- | --- |
|  | *t*-value | P-value | *t*-value | P-value | *t*-value | P-value |
| -6.0 | 0.55 | 1 | -0.33 | 1 | -0.77 | 1 |
| -5.5 | 0.3 | 1 | -1.1 | 1 | -0.9 | 1 |
| -5.0 | 0.33 | 1 | -4.05 | **0.0038 | -2.52 | 0.88 |
| -4.5 | 0.35 | 1 | -3.06 | 0.17 | -2 | 1 |
| -4.0 | -0.29 | 1 | -2.31 | 1 | -0.92 | 1 |
| -3.5 | 0.73 | 1 | 1.05 | 1 | -0.22 | 1 |
| -3.0 | -0.23 | 1 | 1.25 | 1 | 0.92 | 1 |
| -2.5 | 1.01 | 1 | 4.7 | ***1.9x10^-4^ | 1.43 | 1 |
| -2.0 | 0.87 | 1 | 2.78 | 0.40 | 0.57 | 1 |
| -1.5 | 0.69 | 1 | 3.31 | 0.70 | 1.03 | 1 |
| -1.0 | 0 | 1 | 2.82 | 0.36 | 1.5 | 1 |
| -0.5 | 0.63 | 1 | 3.51 | *0.034 | 1.2 | 1 |
| 0.0 | 0.94 | 1 | -0.1 | 1 | -1.05 | 1 |
| 0.5 | -2.12 | 1 | -4.48 | ***5.5x10^-4^ | -0.14 | 1 |
| 1.0 | -2.37 | 1 | -4.33 | *0.0011 | 0.21 | 1 |
| 1.5 | -2.09 | 1 | -2.04 | 1 | 1.13 | 1 |
| 2.0 | -0.92 | 1 | 2.43 | 1 | 2.28 | 1 |
| 2.5 | -2.8 | 0.39 | 2.6 | 0.70 | 4.36 | ***9.8x10^-4^ |
| 3.0 | -2.78 | 0.41 | 2.99 | 0.21 | 4.55 | *4.0x10^-4^ |
| 3.5 | -2.04 | 1 | 2.09 | 1 | 3.29 | 0.075 |
| 4.0 | -1.61 | 1 | 3.67 | *0.018 | 3.67 | *0.018 |
| 4.5 | -1.64 | 1 | 2.98 | 0.22 | 3.34 | 0.064 |
| 5.0 | -2.49 | 0.97 | 1.45 | 1 | 3.42 | *0.048 |
| 5.5 | -2.34 | 1 | -0.21 | 1 | 2.37 | 1 |
| 6.0 | -2.66 | 0.59 | -2.21 | 1 | 1.64 | 1 |
