# Supplementary material for: Unique Configurations of Compression and Truncation of Neuronal Activity Underlie l-DOPA–Induced Selection of Motor Patterns in Aplysia
Source: eNeuro. 2017 Oct 24;4(5):ENEURO.0206-17.2017. doi: 10.1523/ENEURO.0206-17.2017 (PMC5654236; doi:10.1523/ENEURO.0206-17.2017)
Supplement: Figure 5-1 [file enu005172435so18.doc]

|  | | Latency^r^  X^2^=144.63  p=8.5x10^-24^ | | Duration^s^  X^2^=217.68  p=1.3x10^-38^ | | Number of spikes^t^  X^2^=133.82  p=1.2x10^-21^ | | Frequency^u^  X^2^=165.54  p=5.4x10^-28^ | |
| --- | --- | --- | --- | --- | --- | --- | --- | --- | --- |
|  |  | Z | P-value | Z | P-value | Z | P-value | Z | P-value |
| prot | LV | -1.03 | 1 | -0.03 | 1 | -0.07 | 1 | 0.1 | 1 |
|  | LH | -2.98 | *0.043 | -1.14 | 1 | 2.14 | 1 | 4.65 | ***5.0x10^-5^ |
|  | VH | -2.36 | 0.276 | -0.79 | 1 | 1.63 | 1 | 3.9 | **0.0015 |
| ret | LV | -2.24 | 0.378 | -6.47 | ***1.5x10^-9^ | -3.83 | **0.0019 | 2.29 | 1 |
|  | LH | -5.12 | ***4.7x10^-6^ | -3.52 | **0.0066 | -3.6 | **0.0048 | -1.45 | 1 |
|  | VH | -5.36 | ***1.4x10^-6^ | -8.95 | ***5.4x10^-18^ | -6.85 | ***1.1x10^-10^ | 1.44 | 1 |
| Rn | LV | -1.76 | 1 | -4.58 | ***6.9x10^-5^ | -3.67 | **0.0036 | 2.18 | 1 |
|  | LH | -2.75 | 0.089 | 1.15 | 1 | 3.24 | *0.018 | 3.88 | **0.0015 |
|  | VH | -3.6 | **0.0048 | -4.4 | ***1.6x10^-4^ | -1.14 | 1 | 4.53 | ***9.0x10^-5^ |
| n2 | LV | -0.93 | 1 | -4.39 | ***1.7x10^-4^ | -1.73 | 1 | 5.36 | ***1.2x10^-6^ |
|  | LH | -4.19 | ***4.1x10^-4^ | -0.53 | 1 | -0.17 | 1 | 1.03 | 1 |
|  | VH | -3.16 | *0.023 | -5.45 | ***7.4x10^-7^ | -2.06 | 1 | 6.52 | ***1.1x10^-9^ |
| n3 | LV | -2.27 | 0.347 | -5.39 | ***1.1x10^-6^ | -3.7 | **0.0033 | 0.61 | 1 |
|  | LH | -2.57 | 0.153 | 0.36 | 1 | 2.37 | 1 | 3.92 | **0.0013 |
|  | VH | -3.97 | **0.0011 | -6.68 | ***3.7x10^-10^ | -3.5 | **0.0069 | 4.7 | ***3.9x10^-5^ |
